# Evidence for faulting and fluid-driven earthquake processes from seismic attenuation variations beneath metropolitan Los Angeles

**DOI:** 10.1038/s41598-024-67872-3

**Published:** 2024-07-30

**Authors:** Chiara Nardoni, Patricia Persaud

**Affiliations:** 1 Department of Geology and Geophysics, Louisiana State University, Baton Rouge, LA USA; 2 Department of Physics and Astronomy, Alma Mater Studiorum Università di Bologna, Bologna, Italy; 3 Department of Geosciences, University of Arizona, Tucson, AZ USA

**Keywords:** Fault imaging, Earthquake swarms, Crustal fluids, Southern california, Seismic attenuation, Natural hazards, Solid Earth sciences, Physics, Civil engineering

## Abstract

Seismicity in the Los Angeles metropolitan area has been primarily attributed to the regional stress loading. Below the urban areas, earthquake sequences have occurred over time showing migration off the faults and providing evidence that secondary processes may be involved in their evolution. Combining high-frequency seismic attenuation with other geophysical observations is a powerful tool for understanding which Earth properties distinguish regions with ongoing seismicity. We develop the first high-resolution 3D seismic attenuation models across the region east of downtown Los Angeles using 5,600 three-component seismograms from local earthquakes recorded by a dense seismic array. We present frequency-dependent peak delay and coda-attenuation tomography as proxies for seismic scattering and absorption, respectively. The scattering models show high sensitivity to the seismicity along some of the major faults, such as the Cucamonga fault and the San Jacinto fault zone, while a channel of low scattering in the basement extends from near the San Andreas fault westward. In the vicinity of the Fontana seismic sequence, high absorption, low scattering, and seismicity migration across a fault network suggest fluid-driven processes. Our attenuation and fault network imaging characterize near-fault zones and rock-fluid properties beneath the study area for future improvements in seismic hazard evaluation.

## Introduction

**Figure 1:**
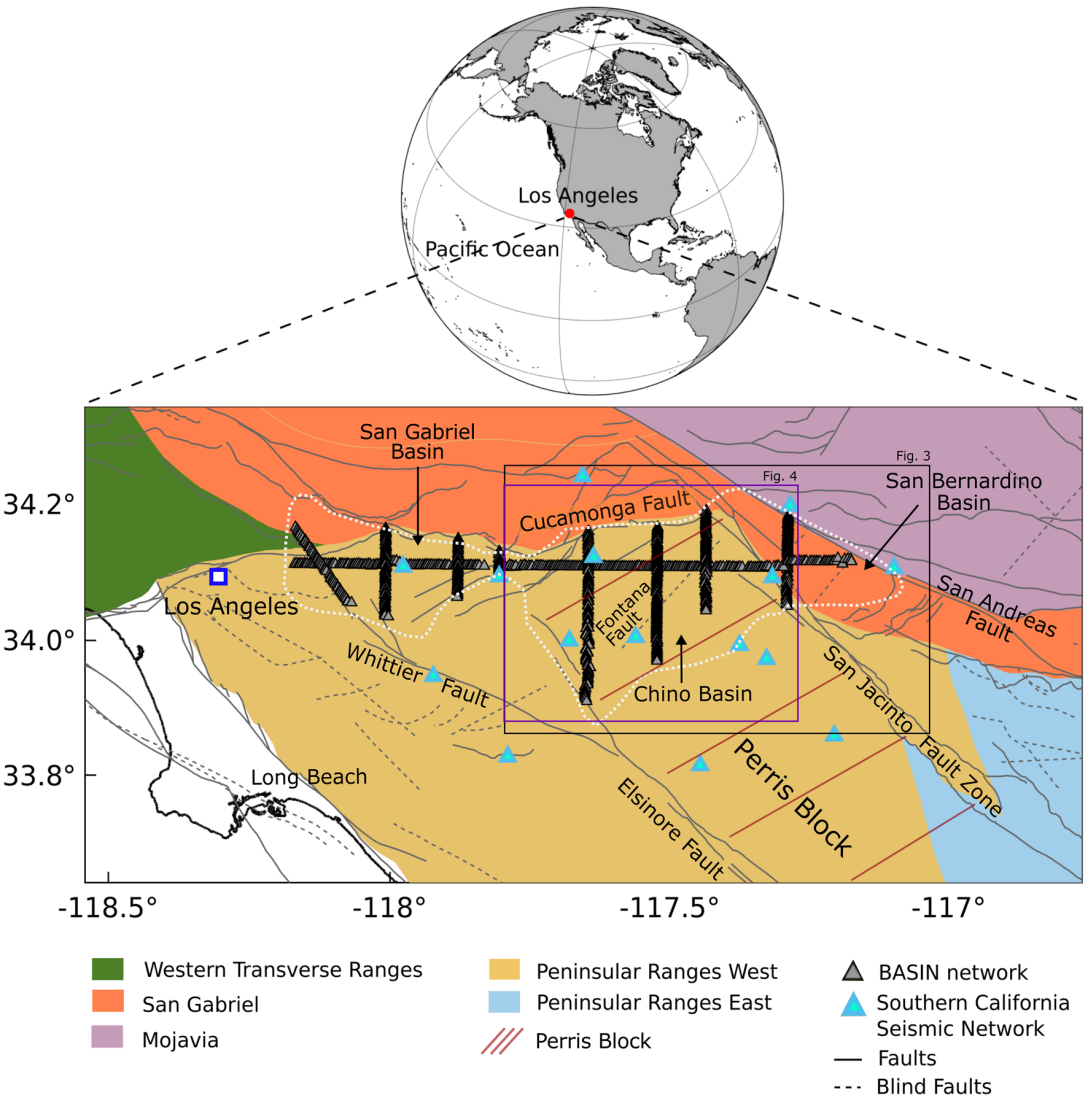
Southern California area, east of Los Angeles: the lithotectonic blocks from the Southern California Earthquake Center Community Rheology Model (CRM)^1^, i.e. Transverse Ranges, Peninsular Ranges, San Gabriel, and Mojavia, are shown with active faults from the Southern California Earthquake Center Community Fault Model (CFM)^2^. The dashed white line indicates the shape of the sedimentary basins comprising the San Gabriel, Chino, and San Bernardino basins. The seismic nodes deployed during the BASIN experiment and broadband stations from the Southern California Seismic Network, used in this study are shown. The extent of the full model domain is shown in Fig. S1. The black and purple polygons indicate the model extents shown in Figs. 3 and 4, respectively .

**Figure 2:**
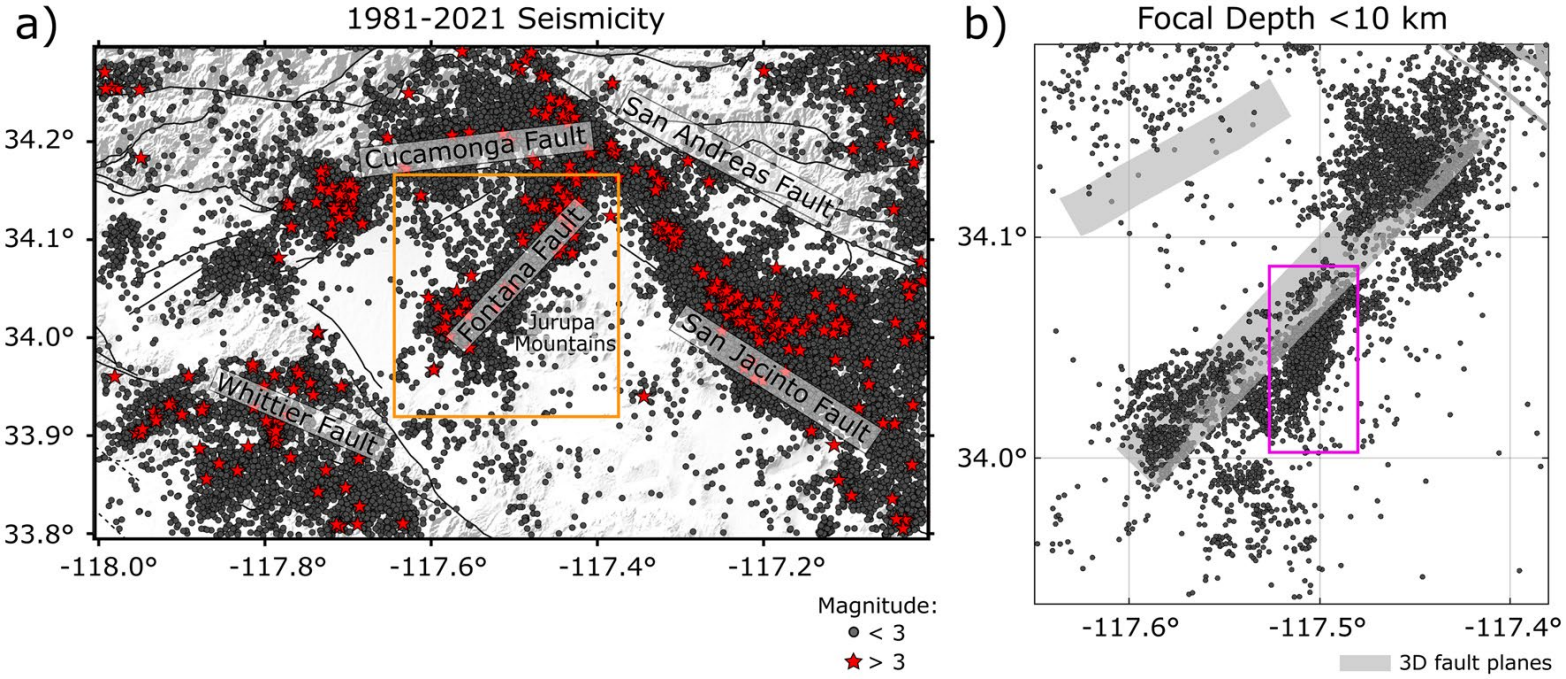
(**a**): Seismicity in the study area (blue rectangle in Fig. S1) since 1981, from the Southern California Seismic Network catalog. The black dots and red stars indicate earthquake magnitudes $< 3$ and $>=3$, respectively. Black lines and gray surfaces are faults from the SCEC CFM 6.1 model. (**b**): Expanded view of the seismicity across the Fontana fault (orange rectangle in panel a) within the upper 10 km of the crust. The magenta rectangle shows the area of the 2019 Fontana earthquake sequence, shown in more detail in Fig. 6.

The densely populated Los Angeles metropolitan area is a region of high seismic risk characterized by interconnected fault systems that can host devastating earthquakes. It is located on top of sedimentary basins that amplify ground shaking and close to the southern San Andreas fault (SAF). Concealed or blind faults crossing the area directly beneath the major population centers have been known to produce unexpected earthquake ruptures such as the 1994 Northridge earthquake (M 6.7)^3^. The region experiences complex active tectonics and is underlain by different crustal blocks that have been distinguished based on their tectonic history, lithology of the rocks, and geophysical properties^4^ (Fig. 1). Abundant small magnitude seismicity is concentrated along the major faults (Fig. 2) and has been primarily attributed to regional tectonic processes.

To determine the physical, social and economic consequences of a major earthquake and avoid the catastrophic impacts of such an event, scientists have modelled a plausible M7.8 scenario earthquake on the southern SAF^5^ and substantial effort has gone into predicting the damage to engineered structures. Despite decades of research to improve estimates of the seismic hazard and determine the variability of ground shaking down to the scale of city blocks, a recent study including information from the surface has shown that the mean hazard ground-motion is overestimated by 65% at a location north of metropolitan Los Angeles and 15 km from the SAF^6^. The overestimation is partly due to state-of-the-art models lacking the geological complexity needed to accurately reproduce earthquake processes and the resulting ground motions, and the sparsity of large earthquake recordings against which these models can be evaluated. The seismic hazard is thus strictly correlated with the local structural complexity that varies across this area. Combining geodetic and geological data allowed an improvement of seismic hazard assessment across the Los Angeles metropolitan region by pointing out the role of conjugate strike-slip faults which seem to accommodate over 50% of the geodetically observed north-south shortening^7^. Walls et al.^7^ showed that the moment release may have been previously overestimated for large faults (reverse and blind thrust faults, such as the Cucamonga fault) and suggested that the seismic moment release is likely to occur as smaller-magnitude events along faults with shorter lengths, punctuated by less-frequent, larger-magnitude earthquakes. Given the importance of integrating diverse observations for identifying areas of maximum stress release (related to fault processes) and seismic wave amplification (related to rock properties), a complete characterization of the large and fine-scale structures is needed to fully understand earthquake rupture processes^8^ and the interaction between faults and predict future earthquake ground motions. For this purpose, we use a new type of seismic experiment to investigate the area east of downtown Los Angeles from the surface and shallow sediments to deeper layers to provide different geophysical observations that allow us to image and model the 3D Earth structure and seismicity evolution near the active faults. During the multi-year Basin Amplification Seismic Investigation (BASIN) experiment^9^, 758 seismic nodes were installed along 10 linear arrays in the San Gabriel, Chino, and San Bernardino basins (outlined with the white dashed line in Fig. 1), in the northern Los Angeles area, to obtain a higher-resolution 3D image of the shallow crust. Several analyses have been completed using the BASIN dataset. Teleseismic receiver functions were computed along the different profiles to map the depth of intermediary sedimentary layers, the sediment-basement interface, and the Moho discontinuity^10–12^ and ambient noise autocorrelations also reveal the shape of the San Gabriel, Chino, and San Bernardino basins^13,14^. Li et al.^15^ produced a new 3D shear wave velocity model of the upper 3–5 km of the sedimentary basins. Villa et al.^16^ took a further step by integrating gravity and seismic measurements to obtain a constrained three-dimensional map of basin depths. In this study, we develop 3D seismic attenuation models that are useful for seismic hazard assessment and use seismic attenuation combined with fault network imaging to investigate the rock properties beneath the study area and further our understanding of their relationship to earthquake processes.

In the framework of seismic hazard assessment, computer simulations of earthquake rupture on the SAF have been used to investigate the wavefield sensitivity to perturbations of the source kinematics and path features and have shown large ground motion amplifications in the sedimentary basins, east of Los Angeles^17^. Wavefield simulations suggest the sedimentary basins east of Los Angeles form an effective waveguide that channels seismic waves into the urban Los Angeles area and produces large energy amplification^18,19^. However, the precise nature of the waveguide has not been identified, yet. A study using ambient seismic noise gives similar results, but shows that wavefield simulations underpredict this amplification effect^20^. Accurately modelling this scenario, especially at the higher frequencies important for engineered systems, requires the implementation of smaller-scale structures into the crustal model. If a low-resolution 3D velocity structure is used, Graves^21^ demonstrates that waveform details are less resolved for the short-period energy. The main challenge of accurate waveform modelling is related to the limited computational resources and knowledge of Earth’s structure. Implementing high-resolution variability of the velocity structure of the Earth laterally and in-depth, especially in the shallow crust, is necessary for reproducing the waveform complexity of the recorded ground motions. A crucial modification is introducing small-scale velocity fluctuations and anelastic attenuation into Earth models to implement realistic scattering and anelastic attenuation in wave propagation simulations^22–24^. A recent study investigating the effects of topography, velocity models, and attenuation on seismic wavefield prediction accuracy in the Salton Trough incorporated an empirical relationship from Olsen et al.^25^ for frequency-independent anelastic attenuation^26^. They demonstrate that including attenuation produces more accurate results, especially at higher frequencies^27^.

Moreover, the potential of seismic attenuation has been proven in imaging Earth features and revealing the processes involved in seismicity evolution^28–30^. The physical mechanisms involved in seismic wave attenuation are scattering due to small-scale velocity fluctuations, and absorption describing the energy loss due to seismic wave propagation through heterogeneous media. As scattering and absorption have shown sensitivity to different features and may show different behaviors in the near-surface and at seismogenic depths^31^, their combined imaging has become a standard approach for a detailed understanding of different processes. Spatial variations of scattering and absorption can be mapped using the peak delay time, defined as the time difference between the S-wave arrival and the maximum amplitude of the envelope^32^, and the attenuation of the envelope of coda waves ($Q_c^{-1}$), which are the wave-trains after the S wave^33^, respectively. At the crustal scale, the measurements of these two seismic attributes allow the identification of lithological contrasts and tectonic interactions, and the mapping of fluid content and sedimentary basins^34–36^. Across faults, imaging both scattering and absorption allows the evaluation of the dynamics of fluid-filled fracture networks and their effects on seismicity distribution^37,38^. High scattering values are indicative of fractured rocks such as across the Vrancea region in Romania^36^ and across fault networks in Southern Italy^37^. Gabrielli et al.^35^ obtained a 3D model of the temporal and spatial variations of scattering across central Italy detecting how thrusts and sedimentary structures control fluid storage, overpressure, and fluid pathways. As shown by Reiss et al.^30^ across a magmatic system, fracture/fault networks can be mapped using peak delay, whereas absorption maps sills, dikes and fluids reservoirs. Given its dependency on heat, deformation, and fluids, absorption has demonstrated remarkable potential in imaging magma, and fluid storage and propagation in tectonic^37^, hydrothermal and volcanic^39,40^ environments. Scattering and absorption models of a shallow hydrothermal system at the Solfatara volcanic crater (Southern Italy) were developed by Di Martino et al.^41^ using data from an active seismic survey. While scattering contrasts mark the main structural features, high absorption anomalies correlate with high soil temperatures and $CO_2$ fluxes associated with fluid-migration pathways. CO_2_-rich fluids have also been detected from attenuation imaging across fault networks in Central Italy^42^ and their role has been proven in the earthquake triggering mechanism as well as in the evolution of the seismic sequences of the Apennines^43^. At the sample scale, peak delay and coda attenuation measurements show sensitivity to the physical properties of the rock, such as pore-space topology, and fractures^44,45^, thus contributing to the calibration of these two seismic attributes.

Previous studies have evaluated frequency-dependent scattering, absorption, and total attenuation across Southern California^46–48^. In addition, Hauksson and Shearer^49^ inferred partially/fully saturated rocks from the shallow crust to seismogenic depths by computing large-scale 3D attenuation models ($Q_S$ and $Q_P$) of the Southern California crust (grid nodes spacing of 15 km). However, the $Q_S$ and $Q_P$ models are frequency-independent and do not distinguish between scattering and intrinsic attenuation. Laboratory measurements on samples of the Orocopia schist which constitutes part of the crust in Southern California show increases in intrinsic attenuation as pore pressure increases^50^ confirming that measurable changes in seismic attenuation can reflect changes in rock-fluid properties. Here, instead of using the regional seismic network only, we take advantage of the densely distributed seismic nodes of the temporary BASIN network (Fig. 1), that, even in a high-seismic noise setting, recorded very low-magnitude earthquakes allowing us to increase our data coverage and model resolution. We develop frequency-dependent 3D scattering and absorption models across the Chino and San Bernardino basins using recordings from eight nodal arrays deployed during the BASIN surveys (recording periods of the arrays in Fig. S1). As mentioned above, while the microseismic activity characterizing the Chino basin is sensitive to changes in regional stress in southern California^51^, the shallow seismicity may also likely reflect the short-term strain transient from fluid movement. As some of the recent seismic sequences and swarms in the area are still unresolved (2019 Fontana sequence^52^), this work aims to image the complexity of the structure by investigating potential fluid pathways along faults and their effects on seismicity evolution, which is a relevant aspect of seismic hazard assessment. We thus include an analysis of the seismicity to obtain fault plane orientations. By combining attenuation and fault network imaging, we identify fault/fracture networks and potential fluid migration across the shallow crust providing complementary observations on seismic source distribution and wave propagation effects.

## Results

**Figure 3:**
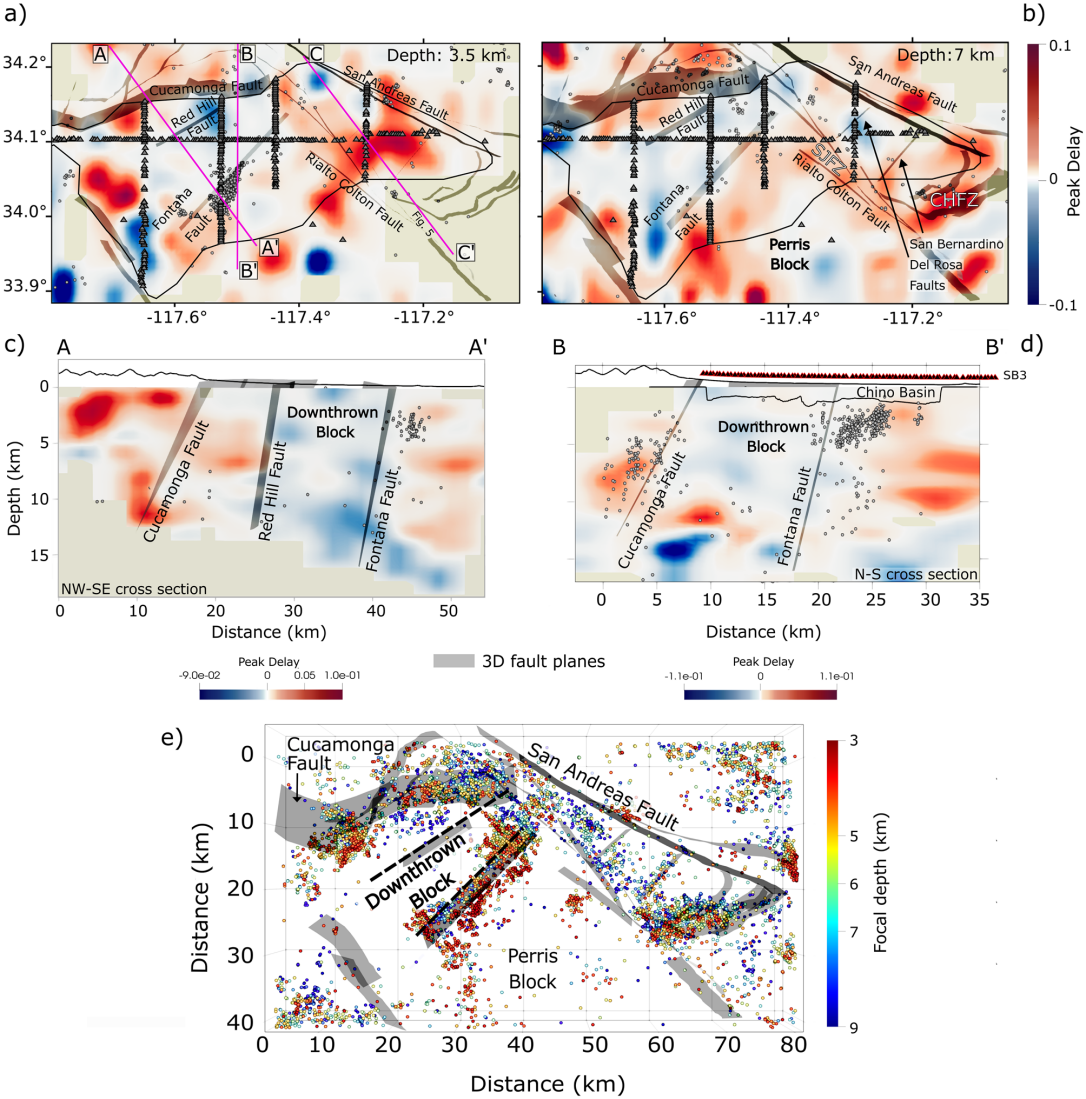
(**a**)–(**b**): Horizontal slices showing scattering anomalies at 3.5 and 7 km depth along with 3D fault planes. The maps show the earthquakes within 1 km depth above the slices taken from the Southern California Seismic Network (SCSN) catalog. We only show earthquakes that occurred during the BASIN experiment. CHFZ and SJFZ indicate the Crafton Hills and San Jacinto Fault zones, respectively. (**c**–**d**): A-A’ and B-B’ cross-sections along the magenta lines shown in panel (a) across the Cucamonga, Red Hill, and Fontana faults. The C-C’ cross-section as indicated in panel (a) across the San Bernardino basin is shown in Figs. S3 and 5. The scattering model is shown for the 18 Hz central frequency band. Results for the 9 Hz frequency band are shown in Fig. S8. (**e**) 3D view of the seismicity distribution between 3 and 9 km from the Hauksson et al.^52^ catalog (1981–2021). The black dashed lines mark the extent of the downthrown block. Small triangles are stations from the BASIN array. 3D gray fault planes are from the SCEC CFM 6.1.

**Figure 4:**
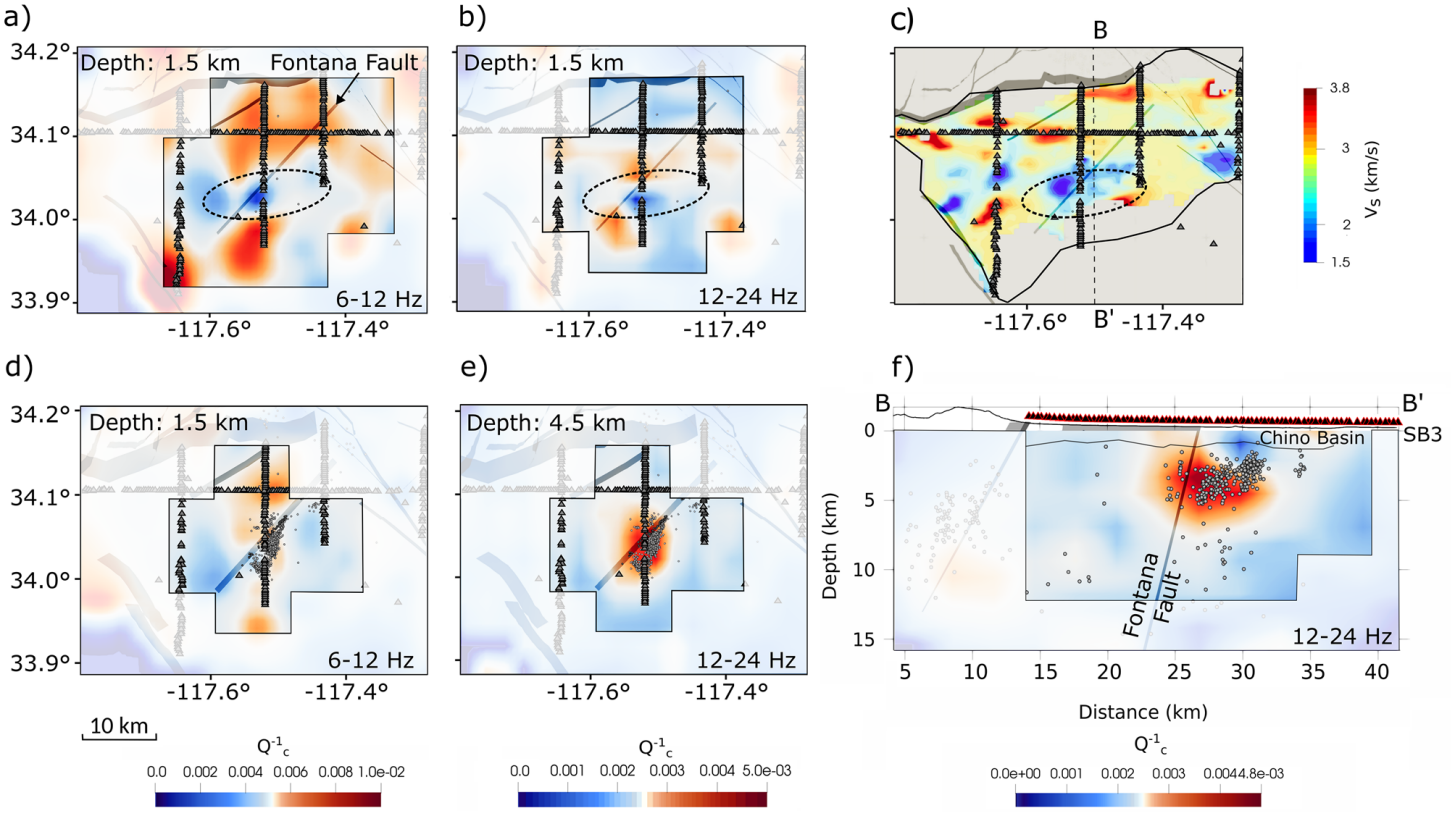
Top panels: depth slice at 1.5 km depth showing the absorption ($Q_c^{-1}$) models at the central frequencies of 9 and 18 Hz (**a**–**b**) and shear wave velocity ($V_s$) variations in the Chino and San Bernardino basins taken from Li et al.^15^ (**c**). The dotted ellipse marks the low-velocity structure and low absorption anomaly. The black polygon in panel c shows the sedimentary basin shape. Bottom panels: depth slice at 4.5 km depth showing the absorption ($Q_c^{-1}$) models at the central frequencies of 9 and 18 Hz (**d**–**e**). Panel (**f**) shows the north-south vertical cross-section B-B’ shown in Fig 3d. The seismicity from the SCSN catalog occurring 1 km above the horizontal slices and within 1 km of the cross-section is shown. Small triangles are stations from the BASIN array. 3D gray fault planes are from the SCEC CFM 6.1.

The attenuation tomography is performed by filtering the seismic waveforms in two frequency bands with central frequencies of 9 and 18 Hz. In Fig. 3a–d, we show the regionalized peak delay (PD) variations at 18 Hz. Low scattering anomalies are shown in blue, while high scattering anomalies are in red for two horizontal slices at 3.5 and 7 km depth, a B-B’ north-south cross-section and a diagonal cross-section, A-A’ (the cross-section C-C’ is shown in Fig. S3 and maps at 1.5 and 4.5 km depth are shown in Fig. S4). In Fig. 4a-b,d-f, we plot the results of the inversion showing the absorption anomalies as the inverse of the coda quality factor, $Q_c^{-1}$ (low absorption in blue, high absorption in red) for two horizontal slices at 1.5 and 4.5 km depth at the two central frequencies, and the B-B’ north-south cross-section at 18 Hz. The earthquake locations and origin times used for the tomography are from the Southern California Seismic Network (SCSN) catalog. For interpreting the absorption anomalies, we limit the area that is resolved in the inversion to 10 km depth and within a 10 km region around the Fontana earthquake sequence, based on the resolution tests (Fig. S5–6). We thus interpret anomalies in a shallow depth slice at 1.5 km and a deeper slice at 4.5 km. In the maps, we include faults obtained from the Southern California Earthquake Center (SCEC) Community Fault Model (CFM 6.1)^2,53^, which are three-dimensional representations of active faults in Southern California. The CFM faults are produced using all available data including seismicity, seismic reflection profiles, well data, geologic cross-sections, and other data types.

**Figure 5:**
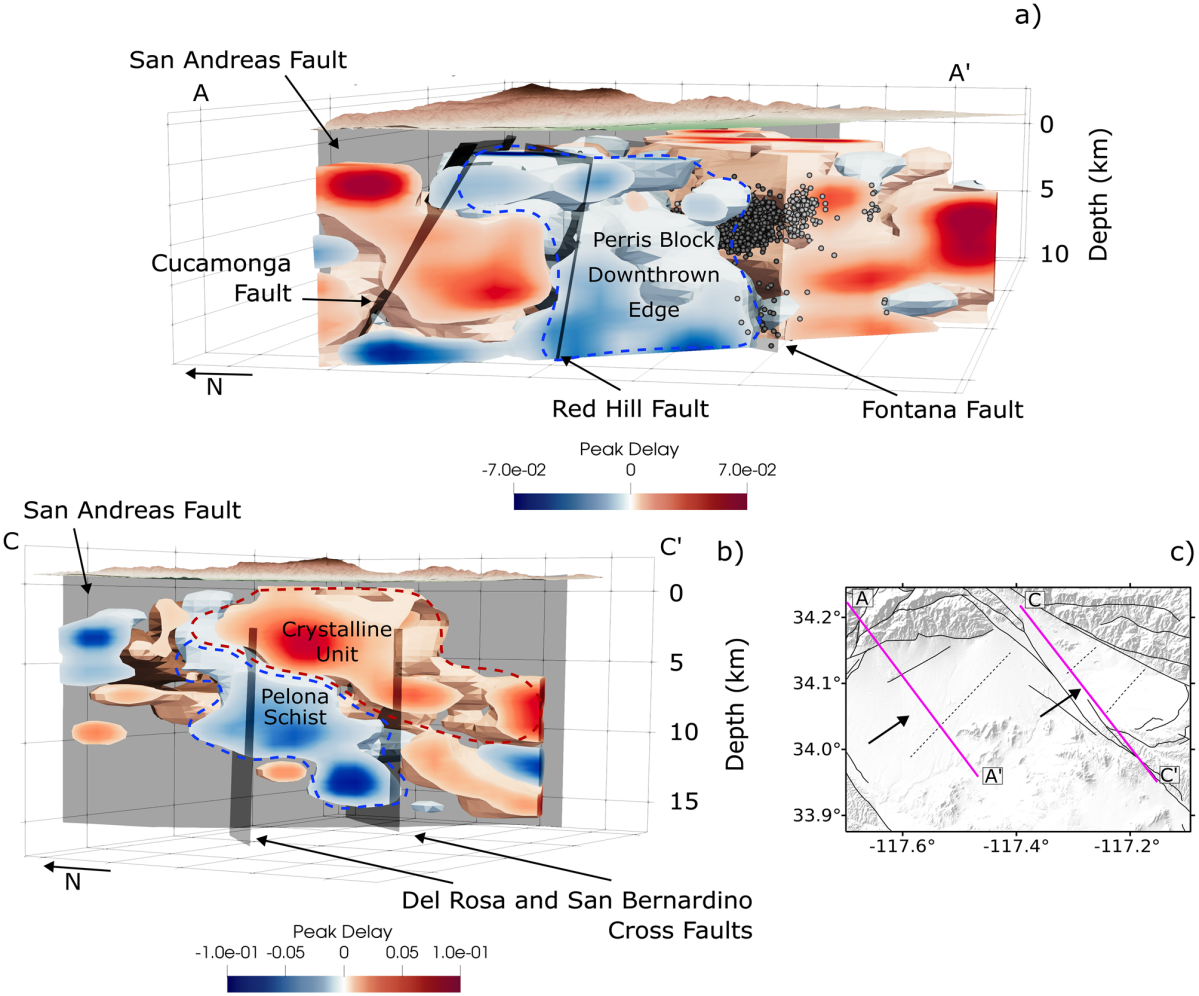
3D views from the west showing the scattering anomalies (12–24 Hz) beneath the Cucamonga-Fontana area (**a**) and in the basement below the San Bernardino basin (**b**). Panel c shows the sections along which the model has been cropped and the view direction. The main fault planes (from the SCEC CFM 6.1) and the seismicity across the Cucamonga and Fontana faults are shown. In panel a, low scattering is associated with the downthrown northern edge of the Perris block suggesting the closure of fractures, while the high scattering volumes correlate with the seismicity distribution (see also Fig. 3e). In panel b, the low scattering anomaly overlain by high scattering marks the lithological contrast between the crystalline rocks and the Pelona schist.

Some of the major faults are characterized by scattering contrasts which mark a distinction between crustal volumes. Figures 3a–d show that the region along the Cucamonga thrust fault is marked by high scattering, where earthquakes are occurring (Fig. 3e), while the region along and north of the Red Hill fault (Red Hill-Etiwanda Avenue fault) extending to the eastern edge of the Cucamonga fault is marked by a scattering contrast in the deeper crust (> 5 km) with low scattering to the south, as also shown in the cross-sections in Figs. 3c–d. At shallow depths, this scattering contrast is mainly localized along the Cucamonga fault. The southern edge of this low scattering volume is consistently bounded by the Fontana fault at all depths (Figs. 3a–d, 5). As also shown in the 3D view of these three faults in Fig 5a, the basement volume between the two prevalently high-scattering anomalies is marked by low scattering values down to 10 km (depth that we consider reliable for interpretation based on hit counts, shown in Fig. S7). This low scattering basement volume is consistent with the same low scattering anomaly found at lower frequencies (central frequency of 9 Hz, Fig. S8). High peak delays also characterize the faults bounding the San Bernardino basin, such as the San Jacinto, and the Rialto-Colton faults, especially at seismogenic depths (7 km depth, Fig. 3b). Across the Crafton Hills fault zone in the southern part of the San Bernardino basin, the hit counts increases at depth and the high scattering remains a dominant feature as shown in Fig. S9. In Fig. S8a–b, lower-frequency (9 Hz) peak delay variations show, on average, high scattering anomalies below the sedimentary basin. The peak delay models also show a horizontal scattering contrast (Figs. 3d and S8c–d), in both frequency bands, within the region bounded by the Cucamonga and Fontana faults around 10 km depth, suggesting potential differences in fracture density. However, this feature lies at the boundary of the well-resolved area and has to be further investigated with improved data coverage. The vertical cross-section crossing the San Bernardino basin and the 3D view of the basement below the basin (Figs. 5b and S3) show a high scattering volume overlying a low scattering volume. This scattering contrast is located around 6–7 km depth suggesting the presence of different crustal rock units.

While the scattering imaging is based on a regionalization approach, the absorption spatial variations are obtained through an inversion and the resolved areas (outlined in the maps with a black polygon) that we can interpret are limited to the Fontana fault region. Figures 4a–b–c compare the shear wave velocity (Vs model from Li et al.^15^) and the absorption models in the two frequency bands at a depth of 1.5 km. The main approximately west-east oriented low $Q_{c}^{-1}$ anomaly across the Fontana fault (latitude 34.0°-34.1° and longitude $-117.6$°-$-117.4$°) coincides with a low $V_S$ anomaly. Figures 4d–e show the depth slices at 4.5 km at 9 and 18 Hz. At lower frequencies, the high absorption anomaly covers part of the region between the Red Hill and the Fontana faults at both 1.5 and 4.5 km depth. At depths near the earthquake sequence, the high absorption anomaly spreads south crossing the seismogenic volume of the 2019 Fontana sequence and is concentrated around the swarm volume at 18 Hz as shown in the cross-section in Fig. 4f, at 3–7 km depth. The north-south extent of the high absorption anomaly matches the orientation of the 2019 Fontana seismic sequence shown in Fig. 4d and the anomaly reaches the surface along and to the north of the Fontana fault. The 4.5 km depth slice of the spike test in Fig. S6 confirms the reliability of this high absorption anomaly associated with the seismic sequence.

**Figure 6:**
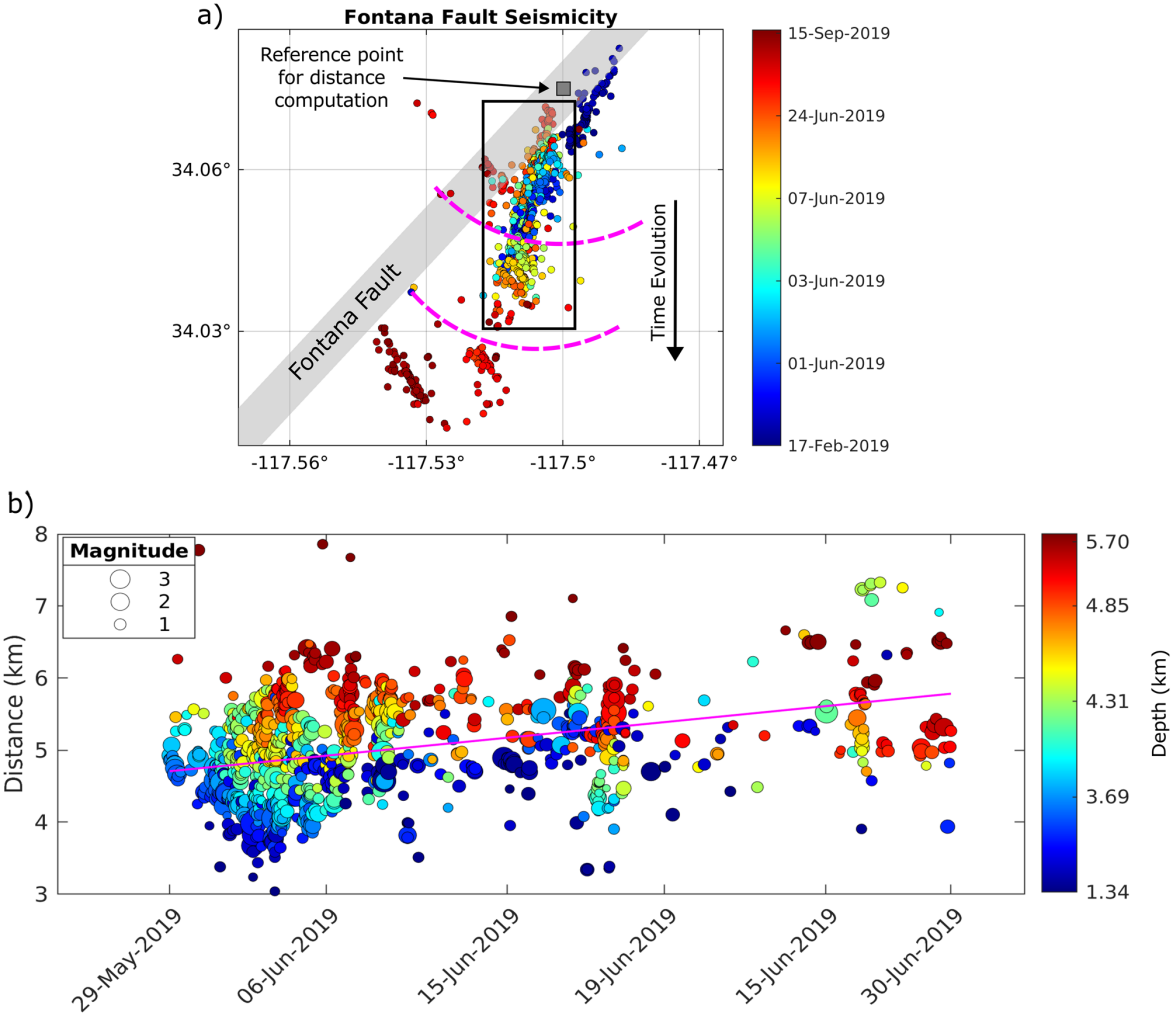
(**a**) Map view of the seismicity that occurred in the Fontana area from February to September 2019 from the 1981–2022 catalog of Hauksson et al.^52^. (**b**) Distance-time evolution of the earthquake swarm in the black polygon in panel (a). Distance is relative to the grey square in (a). The magenta line in (b) is the best fit line. Earthquakes are color-coded based on the date in (a) and the focal depth in (b).

**Figure 7:**
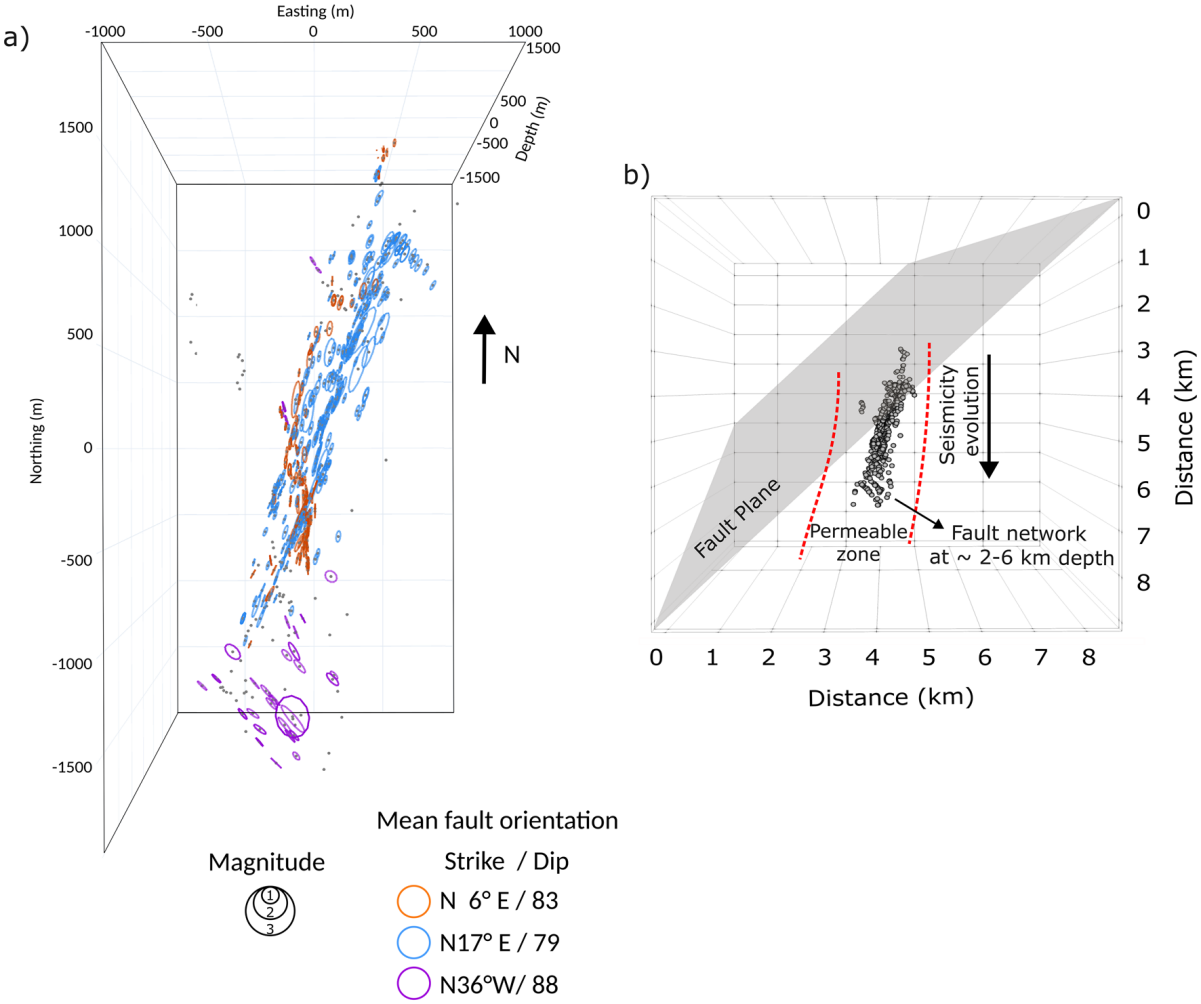
Map view (**a**) of the modelled fault networks corresponding to the 2019 Fontana seismic sequence. We used the seismicity presented in the black rectangle in Fig. 6a. The circles indicate the preferred fault orientation and are colour-coded based on the cluster classification resulting from the analysis. Map view (**b**) of the Fontana fault and seismicity summarizing the findings from the seismicity analysis in Fig. 6 and fault network and absorption imaging. The red dashed lines indicate the region interpreted as permeable based on the absorption model in Fig. 4e.

To investigate the correlation between the attenuation anomalies and the earthquake sequence, we first investigate the spatial and time evolution of the seismicity by computing the distance between the hypocenters and a reference point. Figure 6a shows a map view of the Fontana sequence that occurred from February to September 2019 (we used the relocated catalog from Hauksson et al.^52^). As shown in Fig. S10 in the Supplementary Materials, most of the earthquakes occurred over a short time period (more than 1000 events occurred in June 2019), with some other small clusters over the year. We considered the seismicity (black polygon in Fig. 6a) that occurred in one month (June 2019) and computed the distance between each hypocenter and the grey square in Fig. 6a. The best-fit line in Fig. 6b is obtained with a weighted linear regression where each earthquake is given a weight based on the number of earthquakes within 1 km distance of the event. This approach gives higher weights to earthquakes within the cluster relative to earthquakes located outside the cluster. The hypocenters are described by the linear trend with a correlation coefficient of $\sim$0.5 and a similar trend was obtained using the SCSN catalog (Fig. S11). The trend shows an increase in distance with time that is also reflected in 6a, indicating a roughly north-to-south migration of the seismicity. To provide additional information on the spatial seismicity evolution, the 3D fault plane imaging shown in Fig. 7 and S12 allows for the identification of fault networks, where three main sets of faults are distinguished.

## Discussion

As shown in Fig. 1, the study area is characterized by two main crustal blocks: the San Gabriel block, the central portion of the Transverse Ranges, and the Perris block, which is a structural sub-province of the northern Peninsular Ranges, bounded on the east by the San Jacinto fault, on the north by the Cucamonga fault, and the west by the Elsinore fault. The interaction and convergence between the Transverse Ranges and Peninsular Ranges Provinces resulted in the uplift of the Perris block as well as the San Gabriel Mountains. Previous workers suggest the uplift of the two provinces led to the collapse of the northern end of the Perris block causing the disappearance of an ancient alluvial fan and creating an asymmetric graben bounded on the north by thrust faults of the Cucamonga fault zone, and on the south by one or more unnamed east- or northeast-striking normal faults located between the Jurupa Mountains and the Cucamonga fault zone^54^ (Fig. 1a).

At crustal depths below a few kilometers, scattering attenuation from borehole recordings has shown a local decrease with increasing depth and confining pressure due to the closure of cracks^31^. However, with the exception near the Vincent thrust described below, our scattering models overall show no consistent trend with depth indicating the effects of complex 3D wave propagation which is controlled by the large and small scale tectonic interactions^55^(Fig. 3). Additionally, at low frequencies (0.75 and 1.5 Hz), stations located close to fault zones in Southern California have shown stronger scattering than stations located farther away^47^ which is consistent with our high-frequency scattering models indicating multiple-scales of heterogeneities in the crust. The scattering attenuation pattern shows evidence of a strong influence from the tectonic activity and the past deformation involving distinct crustal blocks^55^. Based on the low scattering volume bounded by the Cucamonga and Red Hill faults on the north and the Fontana fault on the south, we infer that the Perris block collapse occurred along these faults and involved a region we refer to as the downthrown edge of the Perris block. In this scenario, low scattering (Figs. 3a–d,5a) combined with almost absent recent seismicity (i.e., last 40 years) in the upper 10 km of this volume between the Red Hill and Fontana faults (Figs. 2b and 3e) shows evidence of the lower fracture intensity and likely an upper crustal block that has experienced lower strain^45^ (Fig. 5a). Furthermore, we suggest the observed low scattering volume may preserve the wavefront’s high-frequency amplitudes in a channel that extends from near the San Andreas fault westward^17^. In contrast, along the San Jacinto and Crafton Hill fault zones (SJFZ and CHFZ, respectively) bounding the San Bernardino basin, north of the Del Rosa cross fault, and along the San Bernardino cross fault, seismogenic depths (upper 10 km) are characterized by high scattering values (Fig. 3b) which are consistent with the seismicity pattern near these faults (Fig. 3e). However, as shown in Fig. 3e, the recent seismicity is not concentrated across all active faults, some of which are relatively quiet, and does not completely reflect the past deformation. The scattering pattern thus matches some of the major active faults and fractured regions, that host recent seismicity and is also consistent with areas where past deformation occurred.

The basement below the San Bernardino basin consists of two main units: an upper unit of crystalline rocks and a lower unit of Pelona schist which is exposed in the northern part of the San Bernardino basin near 10 km distance in our C-C’ profile^56^. The scattering contrast at 6-7 km depth in Fig. 5b and S3 does not show a clear correlation with the seismicity pattern. However, the high and low scattering volumes mark the lithological difference, indicating that the crystalline rocks and Pelona schist have different attenuation properties: the crystalline rock attenuates more than the Pelona Schist. The Late Cretaceous Vincent thrust separates these two units. In the San Gabriel Mountain area, the Vincent thrust is marked by a km-wide mylonitic zone of metamorphosed greenschist-grade rocks and the underlying Pelona schist has lower seismic velocity^57^ and lower density^56^ than the overlying crystalline unit. The strong scattering contrast across these units likely shows the influence of lithostatic pressure at depth^31^ where more cracks may close in the underlying unit resulting in lower scattering. This observation can be used to identify and map the regional structure of the Vincent thrust.

Within the smaller volume of the absorption models, the results at 9 and 18 Hz show a shallow low absorption anomaly at $\sim 1.5$ km depth in the Chino basin corresponding with an east-west low shear wave velocity ($V_S$) anomaly (Figs. 4a–b–c). Velocity contrasts and interfaces can create reverberations that lower $Q^{-1}$^58^. Our observation suggests that the low-velocity structure mapped in the $V_S$ model^15^ may trap seismic waves and produce reverberations thus lowering coda attenuation. These reverberations occur above the source region of the Fontana swarm. To the north of this region, a shallow high absorption pattern at 1.5 km depth (Fig. 4b-c), close to the sediment-basement interface identified by Villa et al.^16^ at 1-1.5 km depth, likely marks water-bearing sediments. This hypothesis is supported by the presence of several faults which have been identified as groundwater barriers in the Chino Basin, and surrounding area (such as the San Jacinto fault, known locally as Bunker Hill Dike, and the Rialto-Colton fault), and separate the groundwater system into several sub-basins^59^. The surface is underlain by water-bearing sediments that absorb, retain, and transmit the water and groundwater generally flows north-to-south. Modeling of laboratory data has shown that friction on thin cracks and grain boundaries is the dominant mechanism of intrinsic attenuation at most conditions in the Earth’s upper crust and water/fluids have the effect of increasing attenuation^60^. The general attenuation pattern at lower frequencies shows a contrast between the highly attenuating sedimentary basin and the relatively low $Q_C^{-1}$ values in the basement (Figs. 4a and d). However, the patchy behavior of $Q_C^{-1}$ in the basement may still reflect localized zones of fluids, which reduce the friction coefficient within the crust, and reveal the frequency-dependent sensitivity to cracks. High absorption anomalies could thus indicate water entering fractured basement rocks and can influence the effect of the low-scattering block between the Cucamonga/Red Hill and Fontana faults, discussed above, thus reducing seismic wave amplitudes. At higher frequencies, a high absorption anomaly concentrates across the Fontana sequence. The variation in the absorption pattern with frequency marks heterogeneities of different sizes^61^. While at higher frequencies, smaller fractures can be mapped, lower-frequency high-absorption should depict larger water-filled structures. We thus infer that the small fault and fracture network due to the sequence modified the way fluids permeate along the fault. Furthermore, at 3.5 km depth, Figures 3a and d also show a low scattering anomaly located across the sequence volume that correlates with the high absorption anomaly, suggesting fluids percolating at depth. As demonstrated by experimental studies^44^, the scattering process is mainly controlled by the pore size and distribution, and low scattering values thus mark less fractured and heterogeneous rocks. If fluids percolate into the rocks, the pore pressure can increase and rocks compact lowering scattering^35–37^.

As shown in Fig. 2, the Fontana region has been characterized by abundant seismicity over time and recent seismic sequences such as the 2019 sequence shown in Fig. 6a. While peak delay reflects different rock properties and fractures, considering the groundwater system in this area, high absorption may suggest shallow groundwater percolating deeper into the basement and gives a hint to investigate possible driven mechanisms of the sequence. Although the 2019 sequence has not been previously investigated in detail, the overall seismicity in this area has been interpreted in relation to the change in regional stress due to the tectonic loading of the nearby San Jacinto and San Andreas faults^51^. Investigating the sequence’s time evolution (Fig. 6b) points out that it occurs in the shallow crust and close to the fault in the first few days of June and moves slightly towards the north. Then the earthquakes move off the primary fault plane to the south over time. The 3D fault imaging adds crucial information about the sequence, showing the complexity and interconnectedness of the fault geometries. Figures 7a and S12 show that there is one main planar feature where the earthquakes of this sequence are distributed, and it crosses the primary Fontana fault as shown in the seismicity distribution in Fig. 6a. The seismicity starts along this zone (represented by blue circles in Fig. 7) as illustrated by the cluster’s time evolution (Fig. S12). Then another smaller plane of similar orientation is detected more to the south that consists of earthquakes occurring later in June (orange circles). One last cluster, occurring at the end of June (Fig. S12b), is located to the south of the whole sequence and is characterized by cross-faults, at 53° to the main zone of faulting (purple circles in Fig. 7).

Imaging the 3D fault planes demonstrates that the Fontana sequence is characterized by complex fault and fracture networks evolving through time which may be considered part of the larger Fontana fault zone. Such voluminous fault zones have been documented at the southern end of the San Andreas fault and are thought to occur along many active faults^62^. Several sub-clusters and well-defined planes are recognized during the sequence with only a few events occurring off of the planes and appearing to be randomly distributed (grey dots in Fig. 7). The north-to-south time evolution of the sequence and its correlation with the high absorption anomaly (Fig. 7b), may indicate the migration of pressurized pore fluids through the dense fault networks. Previous studies support the role of fluid diffusion, accompanied by pore fluid pressure change, on hypocenter migration and characteristics^63,64^. Even though swarms could result from tectonic stress, lubrication by the release of fluids can intensify this activity. Our results therefore provide evidence of a likely secondary driving mechanism in addition to the regional stress. Further investigation is required to clarify the fluid role in the sequence generation.

## Conclusion

Our seismic attenuation imaging is used to characterize the crustal structure near active faults beneath the Chino and San Bernardino basins and evaluate the local seismicity pattern in terms of the long-term tectonic processes and short-term shallow fluid migration. The joint analysis of seismic scattering and absorption allows a highly detailed characterization of the crustal properties complementary to other geophysical observations. Scattering models primarily identify variations in fracture density resulting from the tectonic convergence between the Perris block and the eastern San Gabriel Mountains. High scattering is consistent with the seismicity pattern along the Cucamonga thrust fault, the Fontana fault, and the fault systems to the south and east of the San Bernardino basin thus reflecting fine-scale fault and fracture networks. In contrast, the low number of earthquakes located between the Red Hill and Fontana faults in the uppermost 10 km of the crust confirms that the observed low scattering volume indicates less fractured and attenuating (potentially highly-pressurized) basement rocks in a part of the study area where high-frequency seismic waves likely maintain their amplitudes towards downtown Los Angeles. Seismic absorption provides additional features as it shows sensitivity to fluid-filled rocks in the basement indicating potential shallow groundwater movement supported by the migration of the Fontana earthquake sequence. The overall characteristics of the subsurface indicate a heterogeneous pattern of seismic wave attenuation at multiple scales. Accounting for the attenuation properties is thus necessary to obtain a complete understanding of the potential impact of crustal properties such as fracture networks and fluid migration pathways on seismic hazard assessment.

## Data and methods

### Dataset

In this study, we analyzed velocity seismograms from local earthquakes recorded by temporary seismic nodal arrays deployed during the Basin Amplification Seismic Investigation (BASIN) experiment^9^. The BASIN network comprises 10 dense linear nodal arrays (Fig. 1; array labels are shown in Fig. S1) which were deployed in the San Gabriel, Chino, and San Bernardino basins and recorded data between 2017 and 2019. The dense arrays consisted of 14–260 FairfieldNodal ZLand nodes (Fig. S1) with standard 5 Hz 3-component geophones. The in-line node spacing is $\sim$250 m. From the Southern California Seismic Network (SCSN) catalog (available at https://doi.org/10.7909/C3WD3xH1), we selected earthquakes recorded by the BASIN network with local magnitudes ($M_L$) ranging from 0.3 to 3.6, and focal depths between 0.5 km (a.s.l) and 18 km (b.s.l). The selected events are located between 33.8° and 34.3° latitude and $-117$° and $-118$° longitude. A uniform and dense distribution of seismic stations and earthquakes is crucial for testing the reliability of imaging results. However, most of the events are located near the Fontana fault and the San Jacinto fault zone, and the BASIN nodes were deployed in linear arrays to perform receiver function analysis^12^. To improve the ray coverage outside the BASIN array, we thus include 15 broadband stations shown in Fig. 1 from the regional Southern California Seismic Network (SCSN; https://doi.org/10.7914/SN/CI). The S-wave arrivals were picked using an STA/LTA algorithm and manually checked on each three-component seismogram of the final dataset. Waveforms with spikes and early/late arrivals were manually removed. Further selections during the peak delay computation aim at excluding outliers due to inaccurate picks. The target area is densely populated and characterized by a very high noise level (especially anthropogenic noise), and this required a selection of events and data also based on a signal-to-noise ratio (SNR) criterion. To estimate the noise level, a 1 s time window after the earthquake origin time is considered. We visually inspect the seismograms to ensure this time window occurs before the P-wave arrival. We selected seismograms with an SNR higher than three for both the window comprising the coda and S-arrival. The input dataset for the attenuation analysis is composed of 5600 sets of three-component seismograms (i.e., 16800 waveforms). The open-access code MuRAT (Multi-Resolution seismic Attenuation Tomography)(version 3.0 of MuRAT^65^), a Matlab package for seismic attenuation, scattering and absorption 3D tomography using body and coda waves, is used to produce our models. This code has been previously applied in volcanic^30^ and tectonic settings^35–37^. We filtered the seismograms in two frequency bands (6-12 Hz, 12-24 Hz) by applying a Butterworth filter of order 4. In the text, these are referred to by their 9 Hz and 18 Hz central frequencies. A Hilbert transform was applied to compute the envelopes of the seismograms, which were then smoothed with a moving window of duration eight times the inverse of the central frequency^37^.

### Peak-delay, $t_{PD}$

In heterogeneous media, seismic waves are affected by multiple forward scattering which broadens the seismogram. As the peak-delay time is defined as the lag between the P or S-wave onset and the maximum amplitude of the envelope and increases with the source-receiver distance, it provides quantitative information on the strength of multiple scattering in the Earth^32^. In the near field, multiple reflections/reverberations generated by structures with strong impedance contrasts such as low-velocity heterogeneities and faults can influence the peak-delay measurement thus showing sensitivity to features approaching the wavelength of the seismic wave^45^.

The MuRAT code computes the peak delay time as the lag time between the S-wave arrival and the maximum amplitude of the envelope for each filtered seismogram and applies the following equation to fit a linear trend to the values

1 $$\begin{aligned} log_{10}t_{PD}(f)=A(f)+B(f)log_{10}(t_S) \end{aligned}$$

where $t_{PD}(f)$ is the peak-delay time, $t_S$ is the S-wave arrival time and *f* is the central frequency. A and B are the coefficients of the linear regression (Fig. S14). The logarithmic variations of the peak-delay time ($\Delta log_{10}t_{i}(f)$) estimated for each waveform ($t_{PD_i}(f)$) with respect to a theoretical linear trend :

2 $$\begin{aligned} \Delta log_{10}t_{i}(f)=log_{10}t_{PD_i}(f)-log_{10}t_{PD}(f) \end{aligned}$$

can be assumed as a measure of the relative strength of accumulated S wave scattering along each ray path^61^. Positive $\Delta log_{10}t_{i}(f)$ values indicate zones of relatively high-scattering (red), i.e. heterogeneous and fractured crust, while negative values are low-scattering (blue), i.e. compacted/saturated rocks (Fig. S14). Before applying the linear regression (Eq. 1), we exclude peak delay values $<0.05$ s and $>2$ s to ensure the maximum amplitude is due to the wave packet and not to spikes and as a quality control on the S picking. As a measure of the uncertainty, we estimate the standard deviation $\sigma$ and consider values with 95% confidence (Fig. S14). We map the spatial variations of scattering attenuation from the logarithmic deviation of the S-wave peak delay time (Eq. 2). The peak delay has been described assuming that the waves propagate mostly in the forward direction between the source and receiver based on a multiple forward scattering model^61^. Rock scale calibration has proved that fractures and shear zones strongly affect the peak delay potentially modifying the scattering sensitivity zone around the ray locally^45,66^. We thus perform a standard regionalization approach for peak delay imaging^34–37^, based on source-receiver raypath sensitivity by averaging the peak delay values of all the rays crossing a grid cell considering that each ray is represented by three-component waveforms. The study area is divided into blocks measuring 3 × 3 km horizontally and 1 km vertically, and we thus allocated to each block the average value of the logarithmic deviation of S-wave peak delay time (Eq. 2). The reliability of peak delay spatial variations is assessed based on the hit count map (Fig. S7 and S9), considering cells crossed by at least 5 rays as well constrained^34^. The code performs ray tracing based on Block et al.^67^, and we input the regional CVM-H 15.1 velocity model^68,69^ obtaining the ray paths shown in Fig. S15.

### Coda attenuation, $Q_c^{-1}$: measurement and imaging

The inverse of the coda quality factor ($Q_c^{-1}$) is used to measure coda attenuation. As described by Aki and Chouet^33^, the energy envelope of seismic coda waves *E*(*t*, *f*) decays as

3 $$\begin{aligned} E(t,f)=S(f)t^{-\alpha } e^{-\frac{2\pi ft}{Q_c}} \end{aligned}$$

where *S*(*f*) is a frequency-dependent source/site term, *f* is the central frequency, *t* is the elapsed time from the origin of the event, and $Q_c^{-1}(f)$ is the inverse of the frequency-dependent coda quality factor. The factor $\alpha$ is a constant that depends on the physical model used to describe coda waves. At long lapse times, coda waves comprise multiple scattered waves and can be modeled using a diffusion process. In this assumption, $\alpha = 3/2$ and $Q_c^{-1}$ is a measurement of seismic absorption $Q_i^{-1}$^70^.

The standard choice for coda attenuation measurements in a diffusive regime is setting the coda onset at $t_w=2t_S$ ($t_S$ is the arrival time of S-waves). Based on the dataset, we selected $t_w=1.5t_S$ preserving the approximation of diffusion and SNR. A least-squares linear fit of the logarithm of $E(t, f)t^{\alpha }$ (see Eq. 3) is performed on the Hilbert transform of the signal to estimate $Q_c^{-1}$ values in the coda time window starting at $t_w=1.5 t_S$ from the origin of the earthquake and lasting for $L_w=4s$ (Fig. S16). Values of $Q_c^{-1}$ are accepted if the correlation coefficient of the linear regression is greater than 0.4. The correlation coefficient is then used to weight the average of the three component values. As shown by Calvet et al.^71^, the choice of coda window should exclude transient (coherent) wave packets occurring at short lapse times and should be consistent for all data to avoid the mixing of early and late coda windows and allow the mapping of stable lateral variations of seismic attenuation. In Fig. S17a, we plotted $Q_c^{-1}$ as a function of the ray length to test our assumption of seismic diffusion in the selected window^71^ and applied a selection considering $Q_c^{-1}$ within $\pm 2\sigma$ with respect the mean value. In this case, multiple scattering is approaching diffusion and there is no decreasing or increasing trend associated with coherent waves. $Q_c^{-1}$ is independent and stable with respect to ray length as also assessed by the computed moving average (Fig. S17b), adhering to the physical understanding of $Q_c^{-1}$. To map seismic absorption in 3D, we defined the same grid as we set for peak delay with node spacing measuring 3 × 3 km horizontally and 1 km vertically, and applied an inversion scheme based on sensitivity kernel functions valid in the multiple scattering regime^30^. To assess the resolved areas, we perform checkerboard tests (Fig. S5). A spike test has also been performed to ensure the reliability of the high absorption anomaly at 4.5 km depth (Fig. S6).

### Fault network modelling

We analysed the 2019 Fontana seismic sequence to investigate the time and spatial evolution of the seismicity. We consider the most recent (1981–2022) earthquake hypocenters relocated by Hauksson et al. using a clustering algorithm and the methods described in Hauksson et al.^52^ available at the Southern California Earthquake Data Center. To model the fault networks, we use the open-access code which performs the hypocenter-based 3D imaging of active faults^72,73^. The user defines how many Monte Carlo iterations are performed and, during each iteration, each hypocenter is randomly shifted within its location errors using a normal distribution. The result consists of point clouds around each initial hypocenter and a subset of hypocenters is obtained by randomly selecting one point for each initial hypocenter. The nearest neighbors of this subset within a defined search radius and search time are extracted and a best-fit plane orientation is calculated using principal component analysis. The developers implemented several criteria to assess the plane-fit robustness, to ensure that the nearest neighbors form an actual plane (not just collinear) and to ensure planarity. Based on the criteria, some of the planes are rejected. The fault planes resulting from all the Monte Carlo simulations are averaged to yield the mean fault-plane orientation shown in Fig. 7. The code performs a model validation based on the focal mechanisms specified in the input file. We thus consider the focal mechanisms solutions provided by Cheng et al.^74^ selecting only focal mechanisms with probability $> 0.5$ that the solution is close to the real one. In the code, a classification scheme that groups the earthquakes based on their fault parameters is also implemented.

### Supplementary Information

Supplementary Figures.

Supplementary Information.

Supplementary Information.

Supplementary Information.

## Data Availability

The earthquake catalogs used in this study and the seismic attenuation models are included as separate files in the Supplementary Materials. The BASIN dataset is available at the EarthScope Seismological Facility for the Advancement of Geoscience (SAGE) Data Management Center https://doi.org/10.7914/SN/6J_2019, https://doi.org/10.7914/SN/4M_2018, and from the authors. The open-access MuRAT, Multi-Resolution seismic Attenuation Tomography code used to produce the seismic attenuation models is available at https://github.com/LucaDeSiena/MuRAT (last accessed 20 March 2024). The open-access code used in the hypocenter-based 3D imaging of active faults is available at https://github.com/sandrotruttmann/hypo_fault_imaging (last accessed 20 March 2024).
