# Supplementary material for: Evidence for faulting and fluid-driven earthquake processes from seismic attenuation variations beneath metropolitan Los Angeles
Source: Sci Rep. 2024 Jul 30;14:17595. doi: 10.1038/s41598-024-67872-3 (PMC11289358; doi:10.1038/s41598-024-67872-3)
Supplement: Supplementary file 1 — Supplementary Figures. [file 41598_2024_67872_MOESM1_ESM.pdf]

Evidence for faulting and fluid-driven earthquake processes from seismic attenuation variations beneath metropolitan Los Angeles

Chiara Nardoni[1,2,*] **and Patricia Persaud**[3,+]

[1]Louisiana State University, Department of Geology and Geophysics, Baton Rouge, LA, USA
[2]Alma Mater Studiorum Università di Bologna, Department of Physics and Astronomy, Bologna, Italy
[3]University of Arizona, Department of Geosciences, Tucson, AZ, USA
[*]chiara.nardoni4@unibo.it
[+]ppersaud@arizona.edu

Supplementary Materials

The earthquake catalogs used in this study and the seismic attenuation models are included as separate files in the Supplementary Materials.

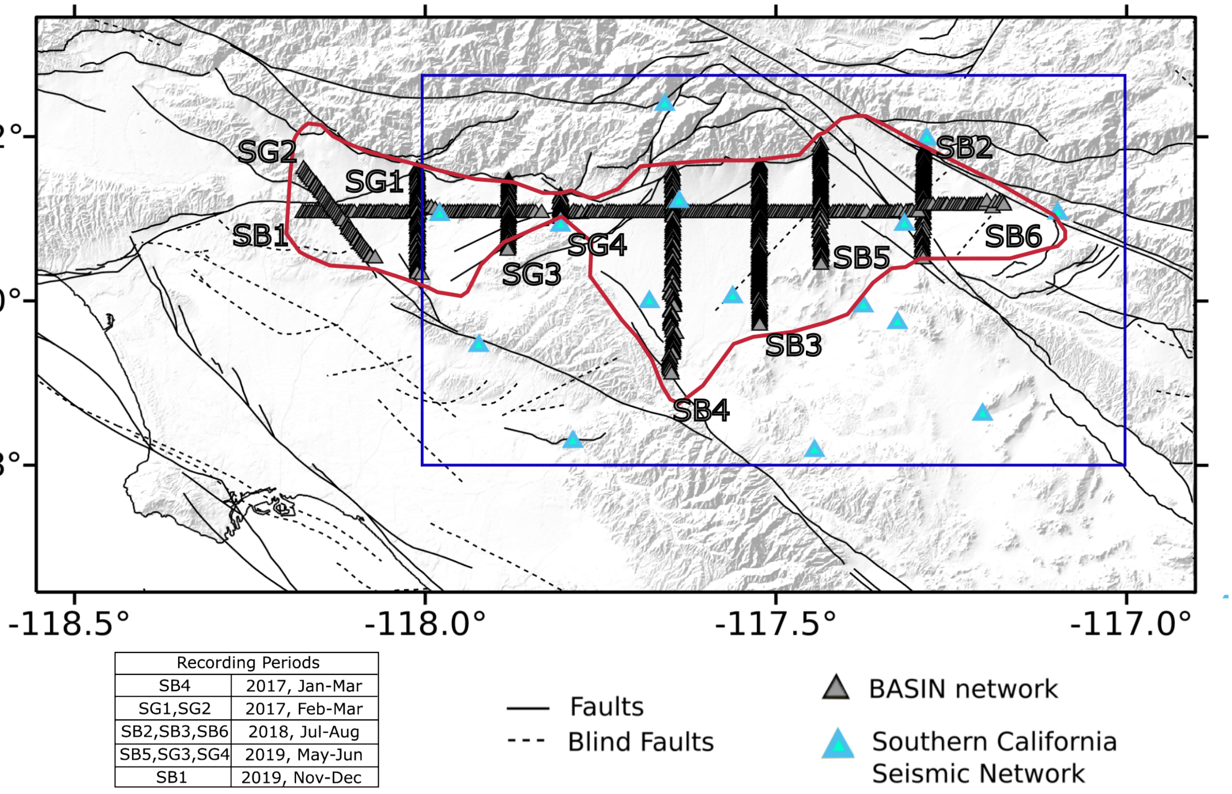

Figure 1. Map showing the locations of the seismic nodes (gray triangles) that were installed along 10 dense linear arrays labelled in the map during the BASIN experiment. The blue rectangle indicates the study area. Eight BASIN arrays (SG3, SG4, part of SB1, SB2, SB3, SB4, SB5, and SB6) were used in this study. The light blue triangles indicate the 15 broadband stations from the Southern California Seismic Network that were included in our dataset. The red line indicates the outline of the sedimentary basins. Faults are from the Southern California Earthquake Center Community Fault Model (CFM) 6.0[1].

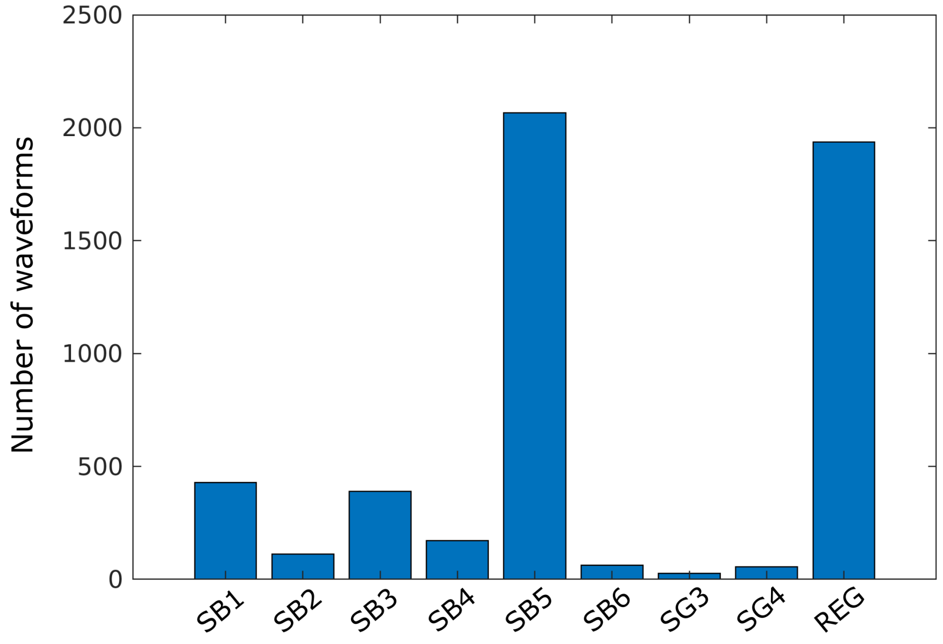

Figure 2. Histogram showing the number of three-component seismograms from each linear nodal array of the BASIN network and the regional network (REG). After the final selection during the peak delay processing, the BASIN network provides 3,315 three-component seismograms out of the total of 5,250.

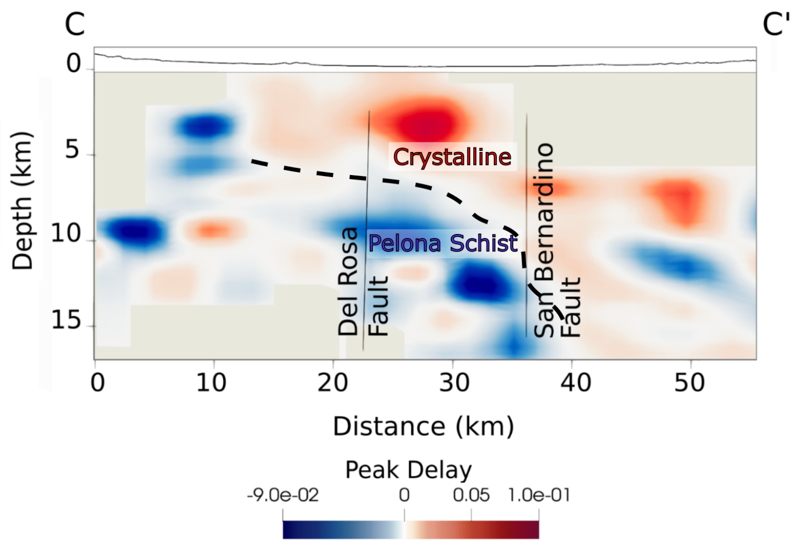

Figure 3. C-C' cross-section of the scattering model at 18 Hz. The location of the cross-section is shown in Fig. 3a.

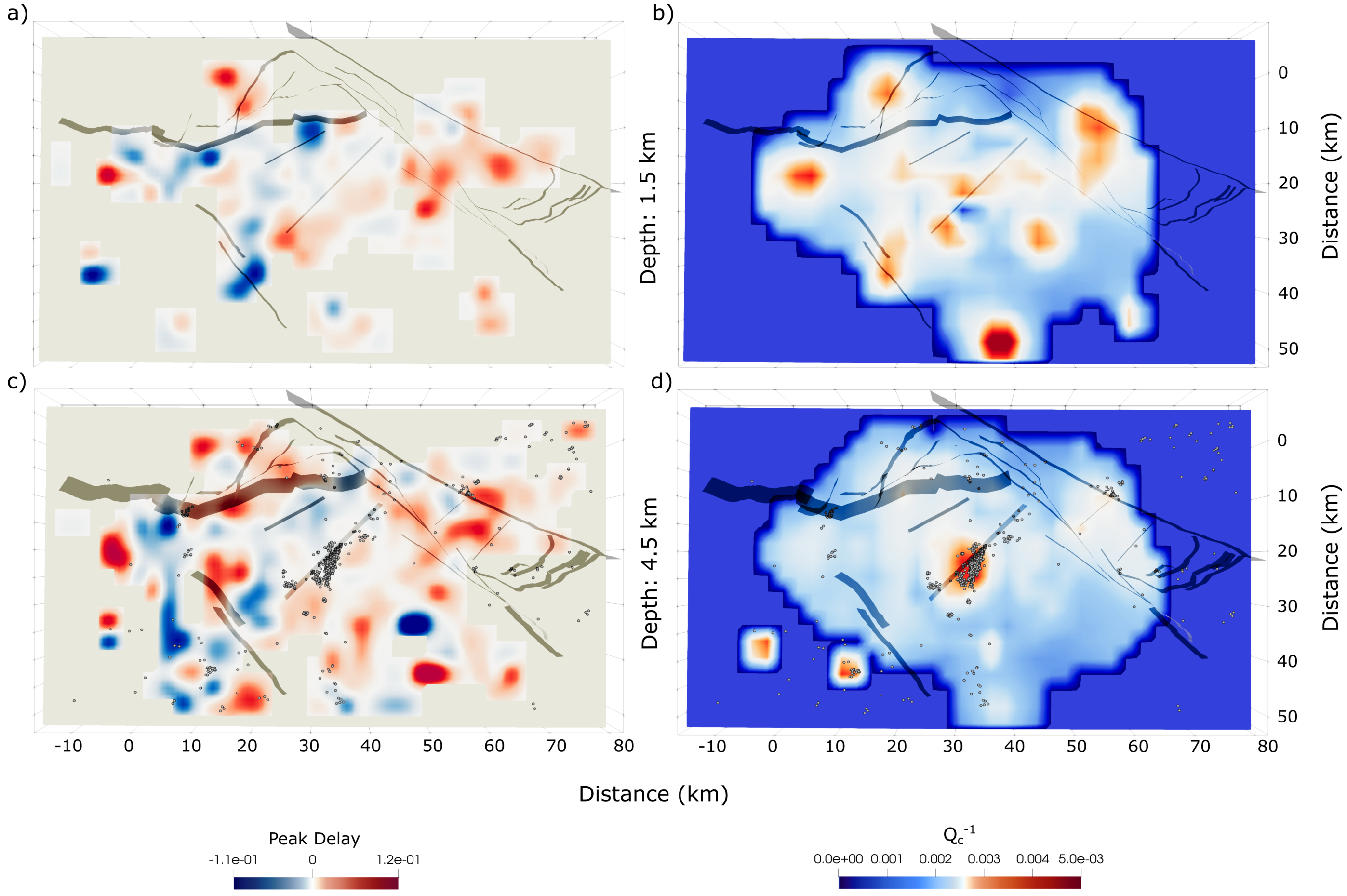

Figure 4. Comparison at 18 Hz between peak delay (left panels) and absorption maps (right panels) at depths of 1.5 km and 4.5 km. Map axes are in units of kilometer.

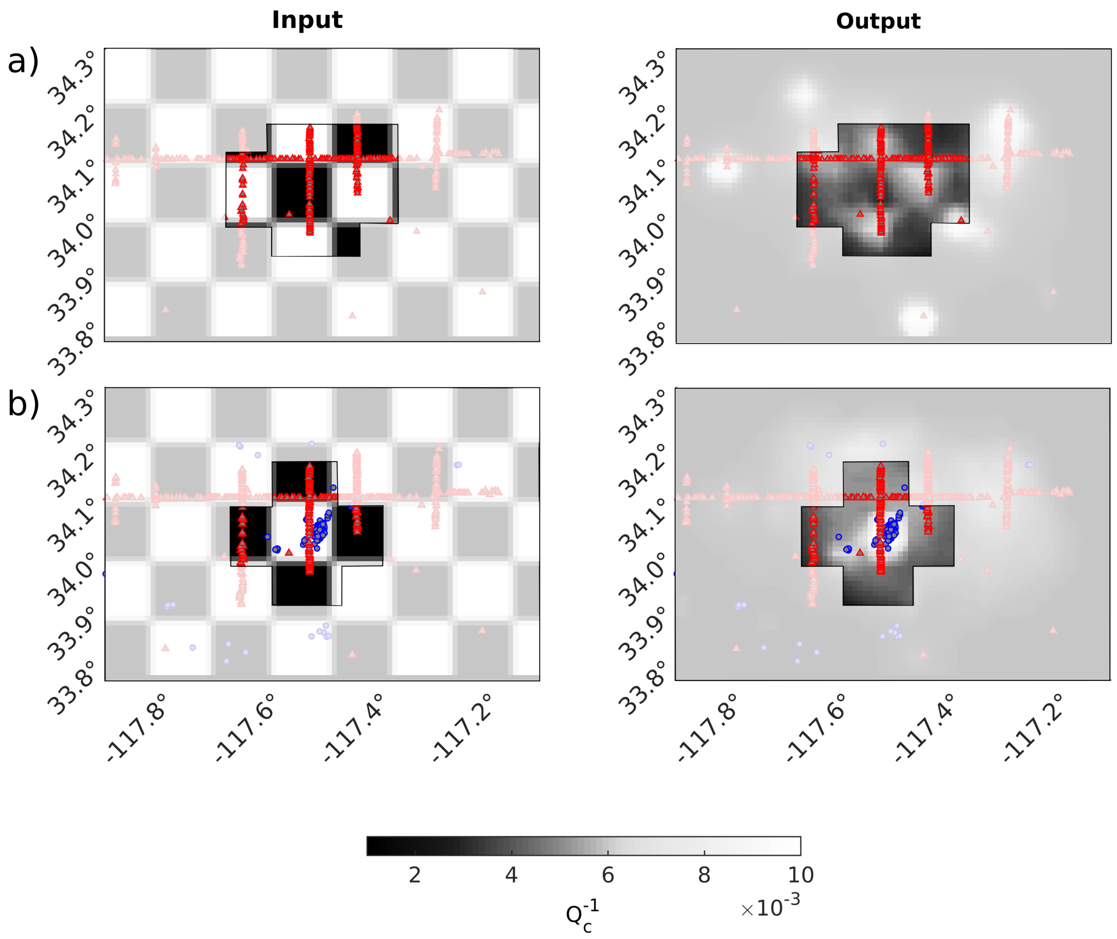

Figure 5. Checkerboard tests (input in the left panels and output in the right panels) for Q_c^{-1} anomalies at (a) 1.5 km and (b) 4.5 km depth at 18 Hz.

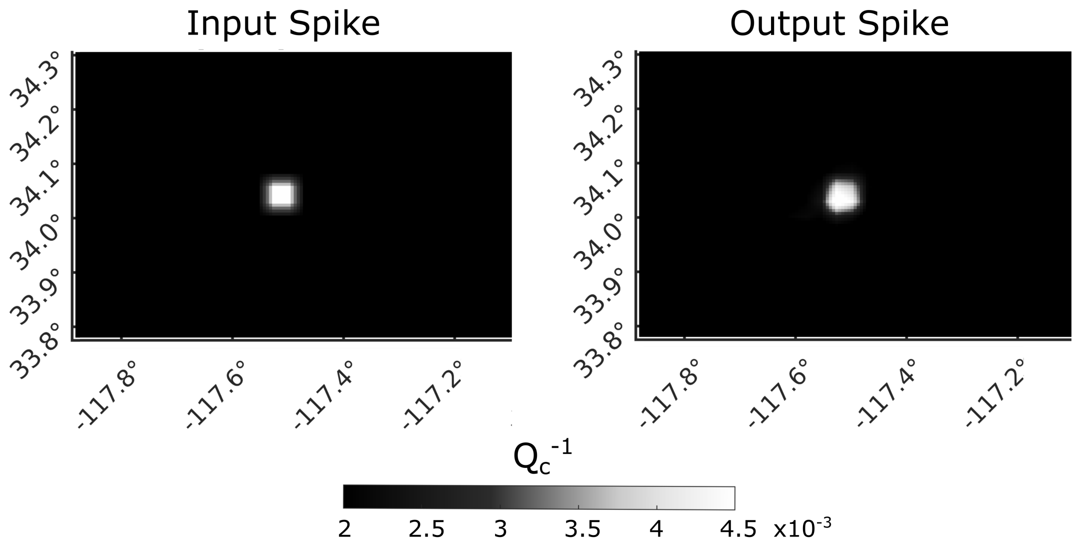

Figure 6. Spike test (input in the left panel and output in the right panel) for a high Q_c^{-1} anomaly at 4.5 km depth at 18 Hz shown in Figure 4e.

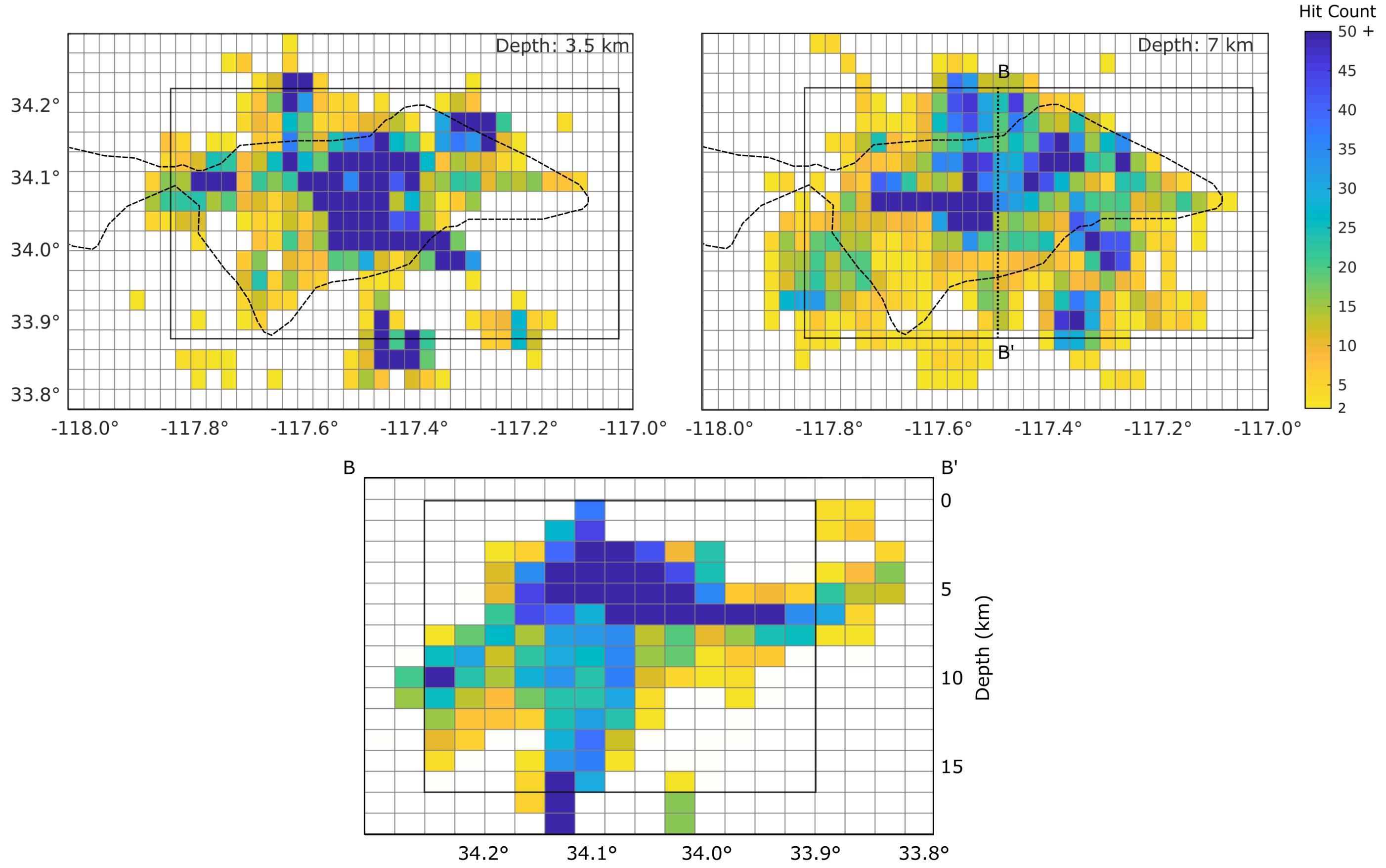

Figure 7. Hit count showing the well-resolved areas of the peak delay maps. Blocks crossed by less than 2 rays are shown in white. The horizontal and vertical slices are taken at 3.5 and 7 km depth, and -117.5° longitude, respectively. The black dashed line shows the outline of the basins. The black rectangles show the extent of the model in Fig. 3. The N-S profile corresponds to profile B-B' in Figure 3a.

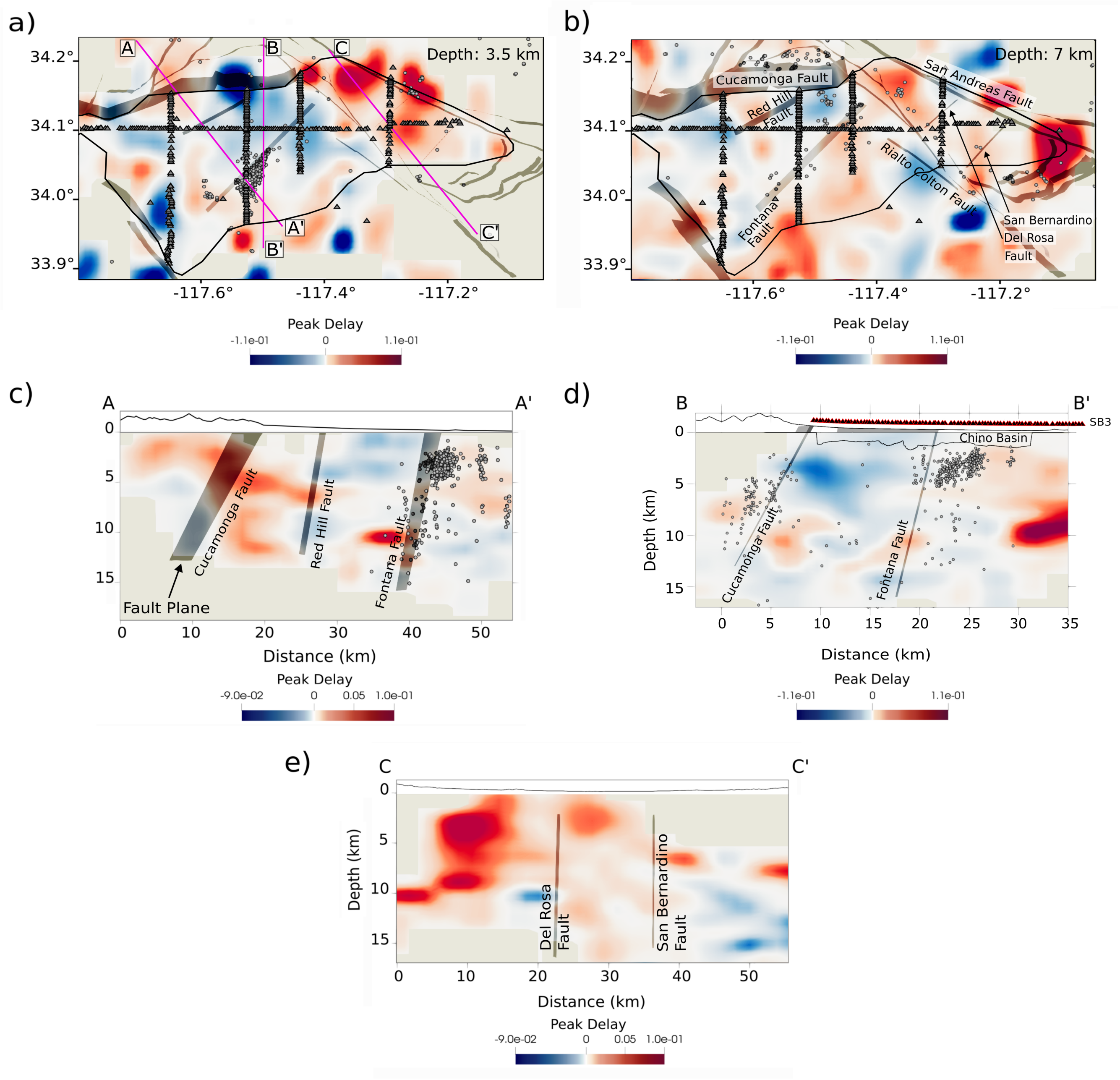

Figure 8. (a)-(b): Horizontal slices showing scattering anomalies at 3.5 and 7 km depth. (c)-(d)-(e): north-south and diagonal cross-sections as shown in panel (a). Results are for the 6-12 Hz frequency band.

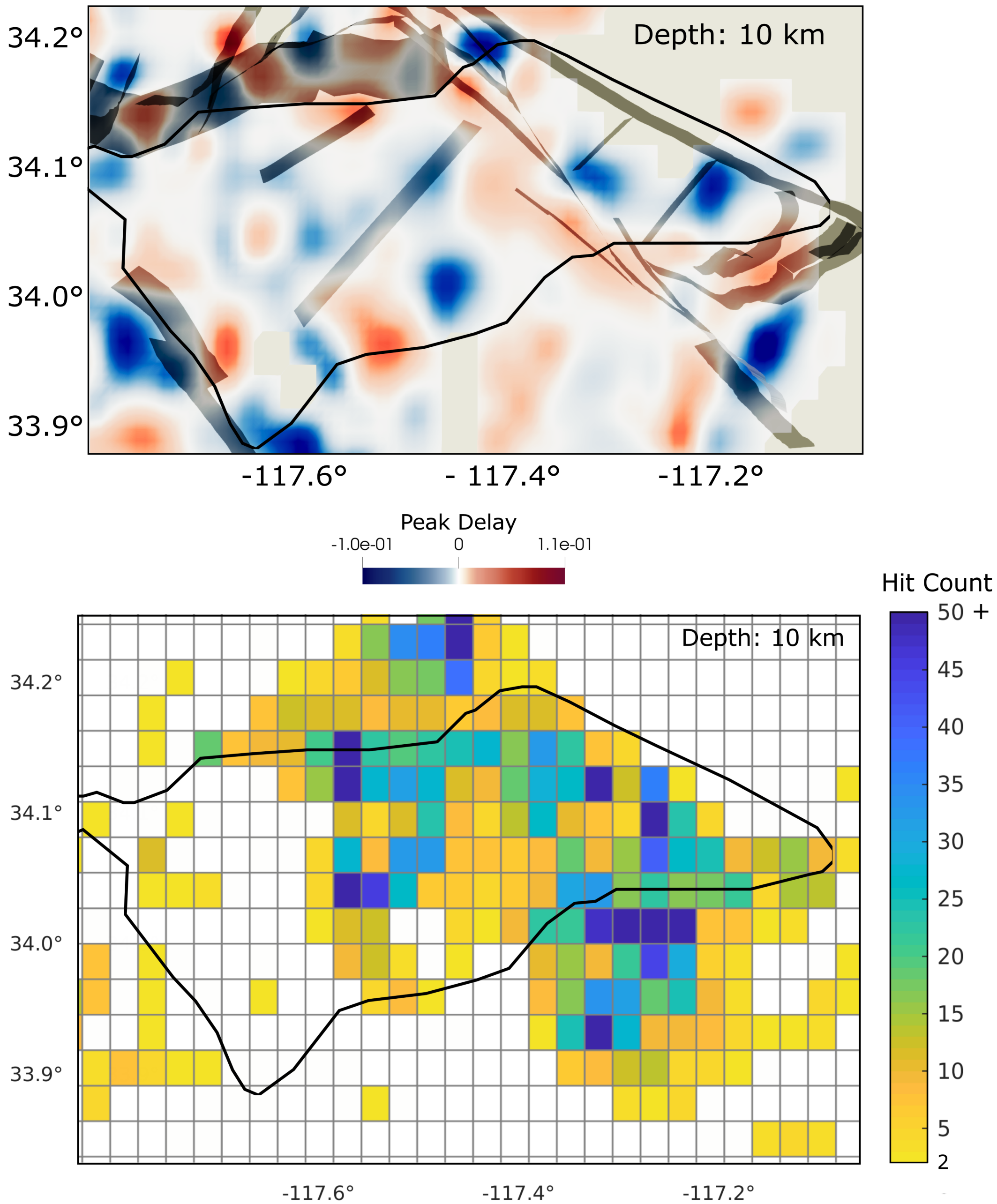

Figure 9. Upper panel: 10 km depth slice of the scattering model at 18 Hz. Bottom panel: hit count map at 10 km depth. Seismicity deeper than 10 km is shown in Fig. S13. The black line shows the basins' outline.

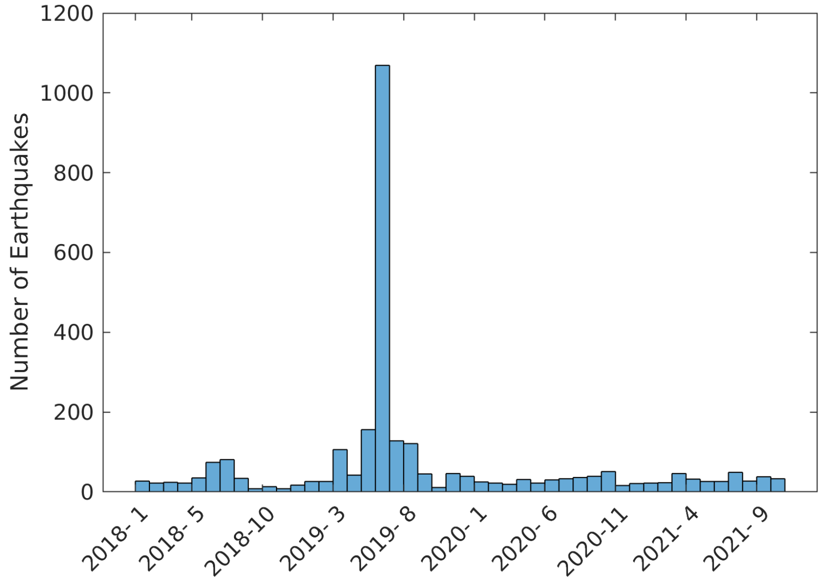

Figure 10. Seismicity in the area of the 2019 Fontana sequence between 2018 and 2021.

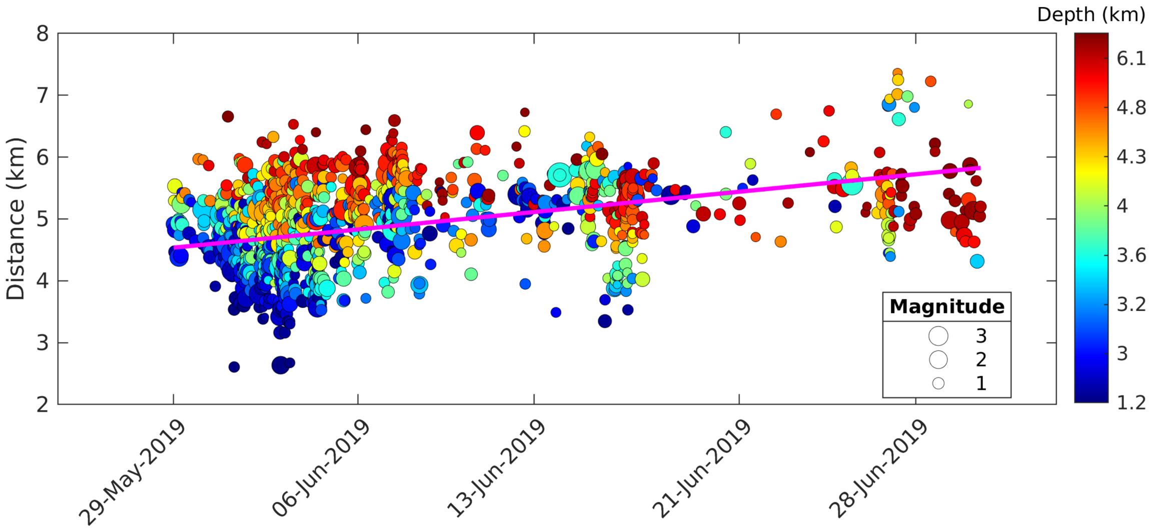

Figure 11. Distance-time evolution of earthquakes that occurred in June 2019 across the Fontana area (black rectangle Fig. 6a), from the SCSN catalog. Distance is relative to the grey square in Fig. 6a. The magenta line is the best fit line. Earthquakes are color-coded based on focal depth.

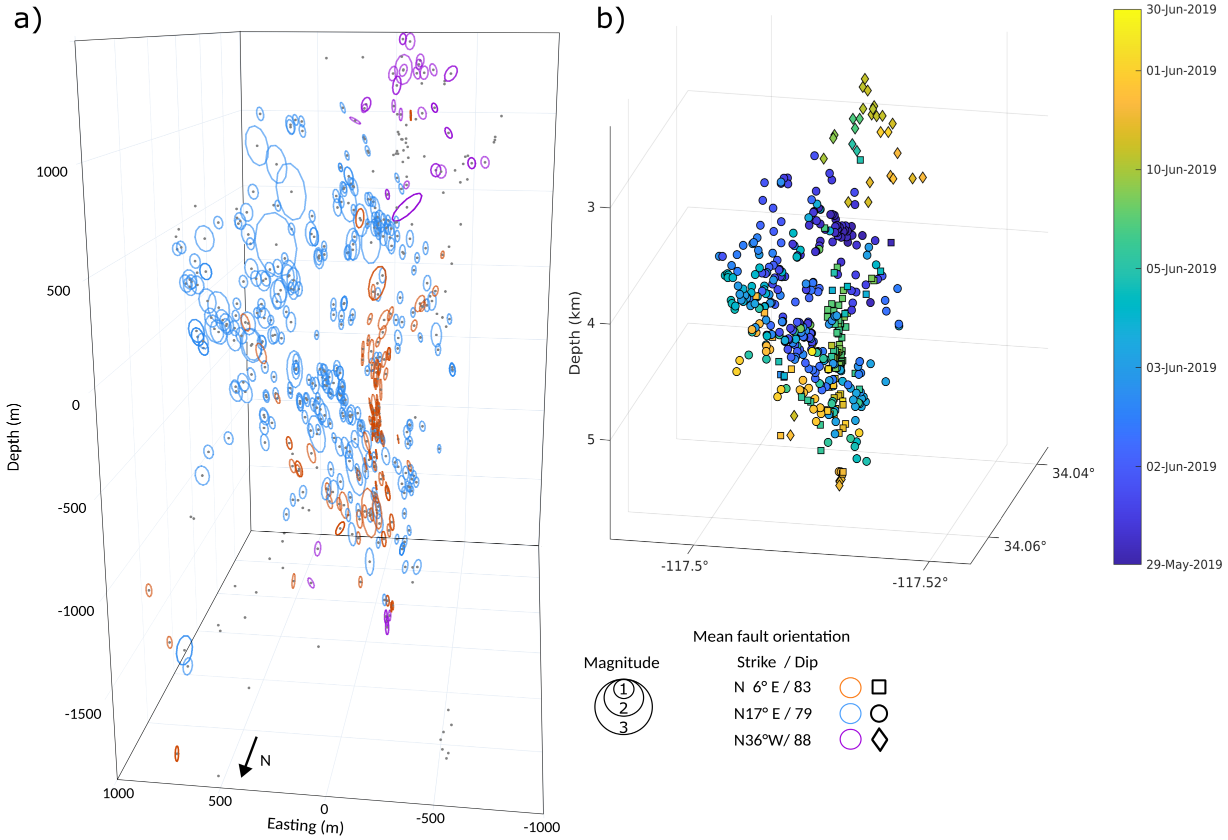

Figure 12. 3D view (a) from the northwest of the modelled fault networks corresponding to the 2019 Fontana seismic sequence. We used the seismicity presented in Fig. 7. The circles indicate the preferred fault orientations and are colour-coded based on the cluster classification resulting from the analysis. 3D view (b) from the northwest of the earthquakes, which are shown with different symbols based on the cluster classification resulting from the 3D fault plane imaging. The symbols are also colour-coded based on the time.

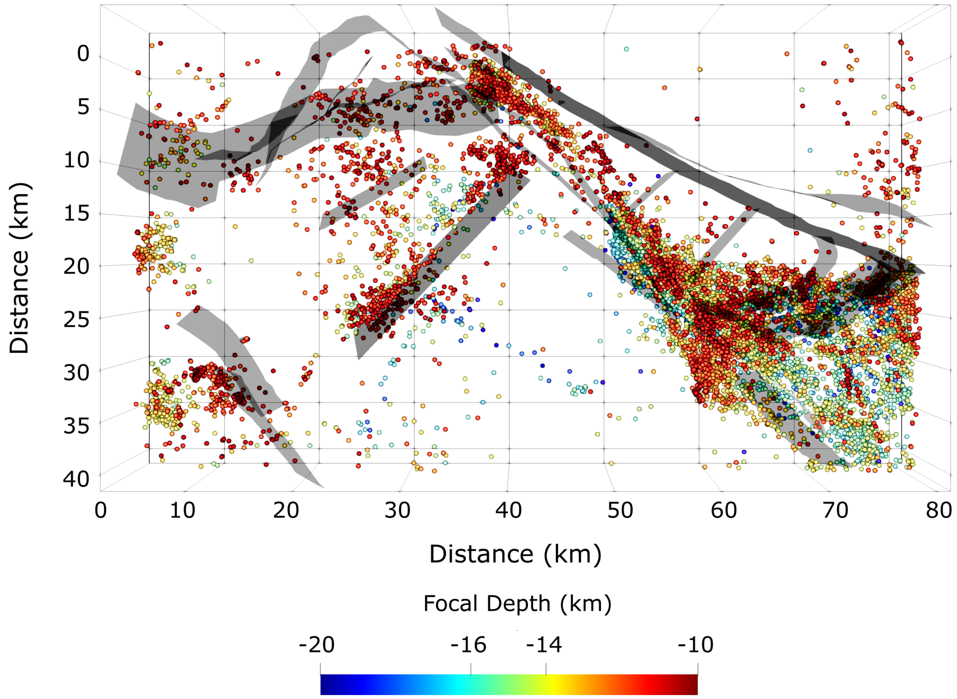

Figure 13. Seismicity (focal depth > 10 km) in the study area from the catalog of Hauksson et al.[2] for the time period 1981 to 2021. Faults are from the Southern California Earthquake Center Community Fault Model (CFM) 6.0.

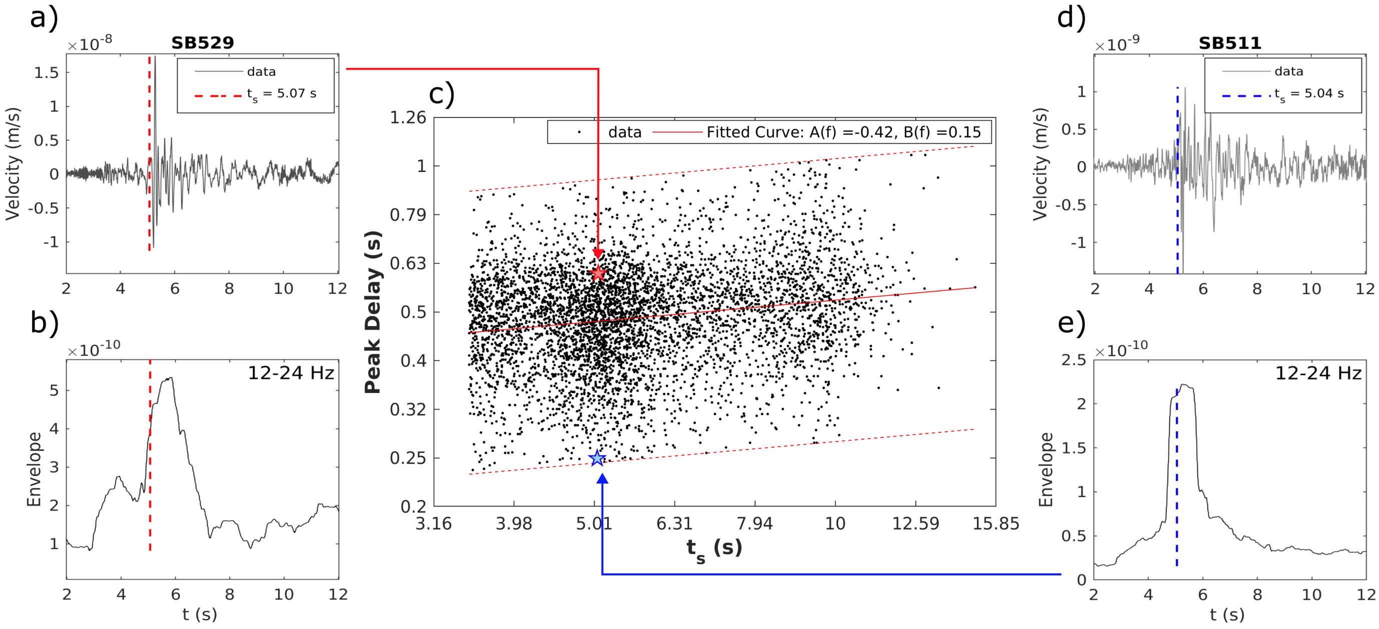

Figure 14. Example of low and high peak delay values for two vertical component velocity seismograms (a and d) recorded at two different seismic stations and characterized by approximately the same S-wave arrival time. (b) and (e): smoothing root-mean-square envelopes in the 12-24 Hz frequency band. The vertical blue and red dashed lines indicate the S-wave onset in the case of low and high peak delay values, respectively. Panel (c): Peak Delay values as a function of the S-wave arrival time. The red line is the result of the linear regression described in the Data and Methods section and the dashed red lines indicate the interval of 2 standard deviations used for selecting the peak delay values. The blue and red stars indicate the position of the low and high peak delay values with respect to the best fit line, respectively. The x- and y-axes labels are shown in a non-logarithmic format to aid the visualization.

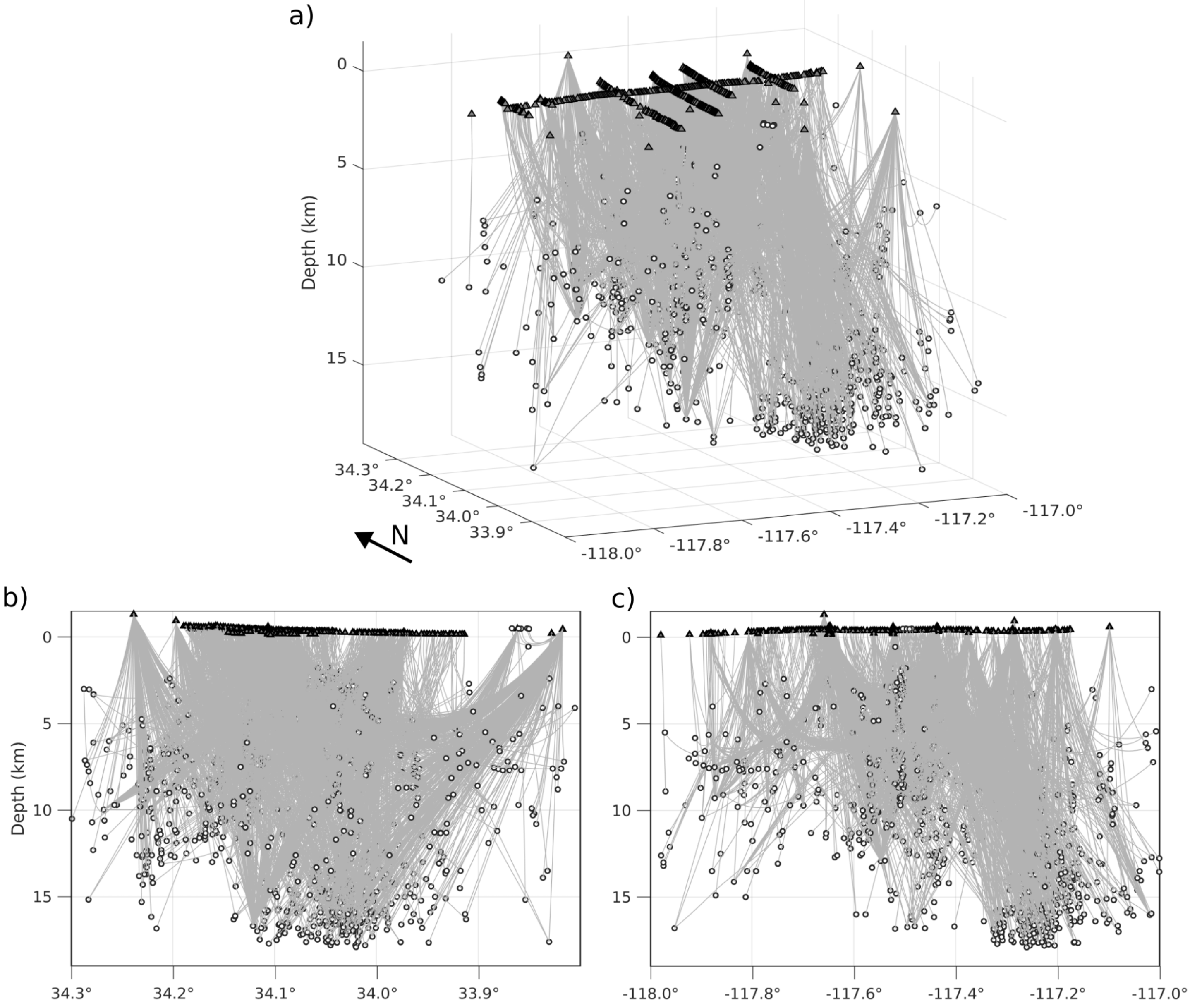

Figure 15. Three-dimensional views of ray path coverage. (a) 3D view from the south-west. (b)-(c) views from the west and south, respectively.

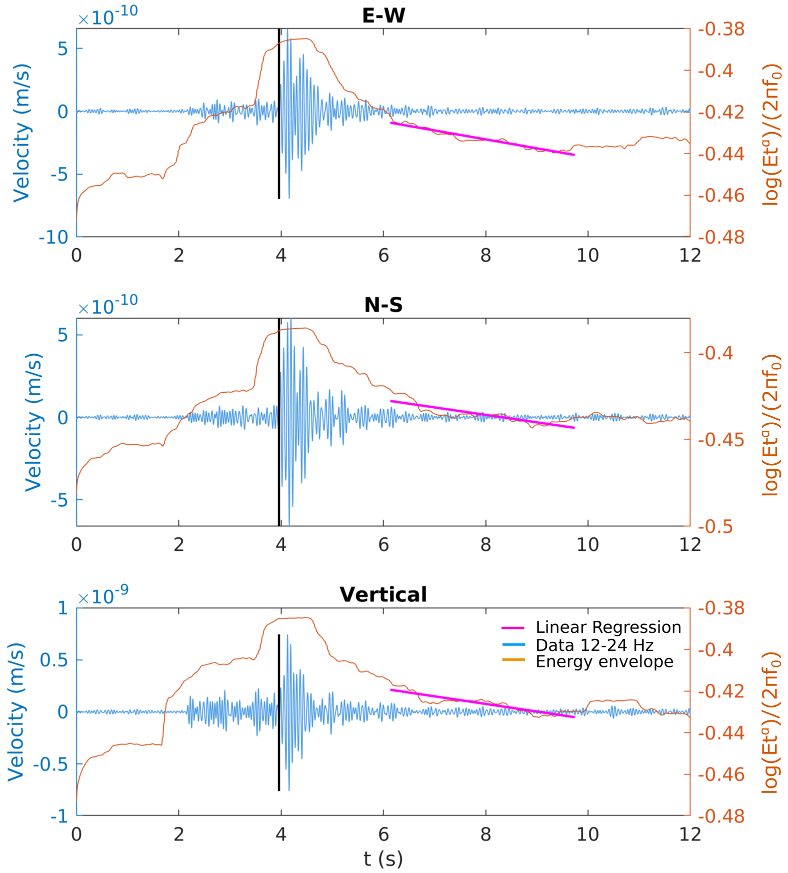

Figure 16. Three component seismograms filtered in the 12-18 Hz frequency band (blue lines) and the energy envelopes ($log(Et^{\alpha})/(2\pi f_0)$, red lines). Best fit line (magenta lines) in the coda time window of length 4 s.

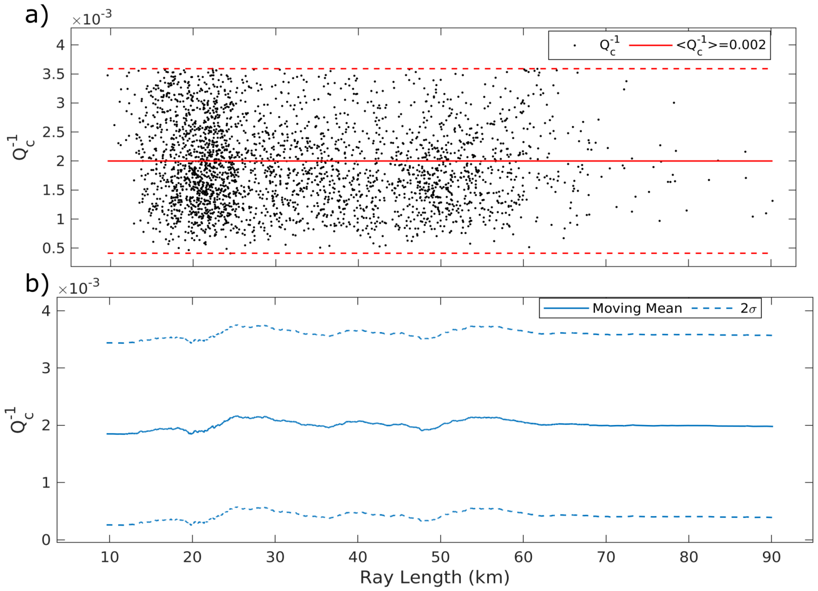

Figure 17. (a) Q_c^{-1} as a function of the raylength at 18 Hz.The red dashed lines correspond to the mean values $Q_c^{-1} \pm 2\sigma$ (σ is the standard deviation). (b) Moving average (blue line) and 2 x standard deviation (dashed blue lines) computed within a moving window of 500 Q_c^{-1} values.

References

1. Marshall, S., Plesch, A. & Shaw, J. SCEC Community Fault Model (CFM), DOI: 10.5281/zenodo.8327463 (2023).

2. Hauksson, E., Yang, W. & Shearer, P. M. Waveform relocated earthquake catalog for southern california (1981 to june 2011). *Bull. Seismol. Soc. Am.* **102**, 2239–2244 (2012).
